# ACC Neuro-over-Connectivity Is Associated with Mathematically Modeled Additional Encoding Operations of Schizophrenia Stroop-Task Performance

**DOI:** 10.3389/fpsyg.2016.01295

**Published:** 2016-09-16

**Authors:** Reggie Taylor, Jean Théberge, Peter C. Williamson, Maria Densmore, Richard W. J. Neufeld

**Affiliations:** ^1^Department of Medical Biophysics, University of Western OntarioLondon, ON, Canada; ^2^Lawson Health Research InstituteLondon, ON, Canada; ^3^Department of Psychiatry, University of Western OntarioLondon, ON, Canada; ^4^Department of Psychology, University of Western OntarioLondon, ON, Canada; ^5^Department of Neuroscience, University of Western OntarioLondon, ON, Canada

**Keywords:** clinical mathematical modeling, schizophrenia encoding, schizophrenia stroop, clinical cognitive neuroscience, schizophrenia neuro-circuitry

## Abstract

Functional magnetic resonance imaging at 7.0 Tesla was undertaken among Schizophrenia participants (Sz), and clinical (major mood disorder; MDD) and healthy controls (HC), during performance of the Stoop task. Stroop conditions included congruent and incongruent word color items, color-only items, and word-only items. Previous modeling results extended to this most widely used selective-attention task. All groups executed item-encoding operations (subprocesses of the item encoding process) at the same rate (performance accuracy being similarly high throughout), thus displaying like processing capacity; Sz participants, however, employed more subprocesses for item completions than did the MDD participants, who in turn used more subprocesses than the HC group. The reduced efficiency in deploying cognitive-workload capacity among the Sz participants was paralleled by more diffuse neuroconnectivity (Blood-Oxygen-Level-Dependent co-activation) with the anterior cingulate cortex (ACC) (Broadman Area 32), spreading away from this encoding-intensive region; and by less evidence of network dissociation across Stroop conditions. Estimates of cognitive work done to accomplish item completion were greater for the Sz participants, as were estimates of entropy in both the modeled trial-latency distribution, and its associated neuro-circuitry. Findings are held to be symptom and assessment significant, and to have potential implications for clinical intervention.

## Introduction

Because of the prominence of thought disorder in the symptom picture of schizophrenia (Sz), performance on cognitive tasks has long been a focus of investigation in the clinical science of this disorder (e.g., Maher, 1966). Deviations in neural circuitry unique to Sz likewise have compelled substantial research attention, at an accelerated rate with contemporary neuroimaging technology (Williamson and Allman, 2011). Mathematical modeling of Sz cognitive performance has indicated cognitive processes spared by the disorder (e.g., scanning and manipulation of material in short-term, or working memory; response-registration processes), and those that are disorder-affected (notably stimulus encoding, or the cognitive transforming of presenting stimulation into a format facilitating collateral processes; reviewed in Neufeld, 2007a) Analytical mathematical modeling moreover has set about identifying spared and affected constituent parameters of disorder-affected processes, and estimating associated neural circuitry through both fMRI (Neufeld et al., 2010), and fMRS (functional Magnetic Resonance Spectroscopy; Taylor et al., 2015).

The Stroop cognitive task, in turn, is arguably the most widely used selective attention task in cognitive science (Eidels et al., 2010). Its ascendant popularity extends to clinical cognitive science (Macleod, 2010), including that directed to cognition in Sz (e.g., Minzenberg et al., 2009). In a typical Stroop task as used in clinical cognitive science, performance requires the naming of a color in which a word is written. Performance is impaired when the named color, and the ink in which the name is written, are mismatched. Recent developments in mathematical modeling of Stroop performance (Eidels et al., 2010) have invited similar analysis of its performance in Sz.

Here we extend previous modeling of Sz cognitive functioning to that on this widely used task. Altogether, we examine if previous formal modeling of Sz cognition also characterizes their Stroop performance. We bring results of Eidels et al. (2010) to bear on findings for the current groups of Sz participants and controls [those with major depressive disorder (MDD) and healthy controls (HC)]. We use a typical clinical-science Stroop paradigm, but one that is extended in light of mathematical Systems Factorial Technology (Eidels et al., 2010). We also examine whether neurocircuitry over-connectivity and its diversion away from encoding-intensive sites in Sz are seen with the present analytically-modeled Stroop task.

### Mathematically modeled cognitive deviation in Sz

Experimental cognitive paradigms, such as those addressing memory and visual search (late- and early-target paradigms; Townsend and Ashby, 1983), have been used to triage processes that are disorder-affected^1^. Convergent experimental evidence and accompanying modeling have implicated stimulus encoding as a Sz-affected process, potentially adversely affecting other processes for which encoding is necessary. Stimulus encoding refers to the conversion of presenting stimulation, such as an alphanumeric probe of a memory-search task, into a cognitive format entering into collateral processes, such as scanning for the probe's presence in a set of previously memorized alphanumeric items (e.g., through memorial template matching). Experimental isolation of this process, and its elongation in Sz, has exploited divergent paradigms (reviewed in Neufeld, 2007a; Neufeld et al., 2010). Symptom significance of this source of cognitive deficit also has been formally explored (e.g., Neufeld et al., 1993; Neufeld, 2007a).

Sources of Sz encoding elongation have been examined using parametric stochastic models (e.g., Neufeld et al., 1993). Modeling results have reliably indicated that the speed of transacting constituent encoding operations (i.e., encoding subprocesses, such as implementing individual alphanumeric features) is spared; the number of subprocesses undertaken, however, is increased. By this account, cognitive workload capacity at the subprocess level is preserved, but efficiency of its deployment has suffered. Using a race horse analogy, running speed is unaffected, but running takes place closer to the outside rail, demanding more strides to complete the extra distance.

Possible contributors to added subprocesses (enumerated in Neufeld et al., 2010) include, for example, initial preparatory activity, that ramps up, or primes the encoding apparatus; Bluhm et al. (2007) have documented abnormal resting-state, intrinsic-network neural circuitry in Sz, a finding compatible with potential stalling of resource recruitment to the service of encoding. Williamson and Allman (2012), moreover, cite evidence for reduced suppression of the default network, potentially exacerbating task-network activation.

The above combination of spared and affected encoding-process parameters can be expressed in selected stochastic latency distributions. One such distribution is the Erlang (e.g., Evans et al., 2000). Its density function is *f* (t) is

$$\begin{array}{l} {f\left(t\right)}_{Erlang}\;=\;\frac{{\left(vt\right)}^{k\prime -1}}{\left(k\prime -1\right)!}v\;e^{-vt} \end{array}$$

with mean

$$\begin{array}{l} {E\left(t\right)}_{Erlang}\;=\;\frac{k\prime }{v} \end{array}$$

and variance,

$$\begin{array}{l} {Var\left(t\right)}_{Erlang}\;=\;\frac{k\prime }{v^{2}} \end{array}$$

where the shape parameter, *k*′, represents the number of subprocesses, and the scale parameter, *v*, represents their rate of completion. The diagnostic status of Sz is associated with increased *k*′, but no difference in *v*. Support for this parametric account has converged from several paradigms and model variants (reviewed in Neufeld et al., 2007a, 2010).

The additional-subprocess account has also been extended (elsewhere) to mixture-model structures, providing for individual differences in values of the above parameters (e.g., Neufeld et al., 2010). For example, mixing the parameter *k*′ on a Poisson distribution with parameter *m*, and mixing the rate parameter, *v*, on a gamma distribution, with scale and shape parameters *r* and *k*, produces a density function,

$$\begin{array}{l} f{\left(t\right)}_{Mixture;Poisson,m\left(k\prime \right);\;Erlang,\;r,k\left(v\right)}\\ =\;\overset{\infty }{\underset{k\prime =1}{\sum }}\frac{m^{k\prime }}{k\prime }e^{-m}\frac{r^{k}\Gamma \;\left(k\prime +k\right)t^{k\prime \;-\;1}}{\Gamma \left(k\right)\left(k\prime \;-\;1\right)!{\left(r+t\right)}^{k+k\prime }} \end{array}$$

the distribution mean being

$$\begin{array}{l} {E\left(T\right)}_{Mixture;Poisson,m\left(k\prime \right);Erlang,r,k\left(v\right)}=\frac{mr}{k-1} \end{array}$$

the expected variance given *k*′ and *v* being

$$\begin{array}{l} E{\left[Var\left(Tk\prime ,v\right)\right]}_{Mixture;Poisson,m\left(k\prime \right);Erlang,r,k\left(v\right)}\;=\;\frac{mr^{2}}{\left(k-1\right)\left(k-2\right)} \end{array}$$

and total variance (i.e., *E*[*Var*(*T*|*k*′, *v*)] + *Var*[*E*(*T*|*k*′*, v*)]) being

$$\begin{array}{l} {Total\;Var\left(Tk\prime ,v\right)}_{Mixture;Poisson,m\left(k\prime \right);Erlang,r,k\left(v\right)}\\ =\frac{mr^{2}\left(2\left(k-1\right)+m\right)}{{\left(k-1\right)}^{2}\left(k-2\right)}. \end{array}$$

Here, the sole parameter to change with the occurrence of Sz diagnostic status is that of *m*, whose increase moves the distribution of *k*′ upward.

A further mixture—this time of *m* via gamma_*R, K*._, allowing for individual differences in a Poisson process that randomly distributes *k*′ over trials of individual performance (e.g., Neufeld et al., 2007b; see also Busemeyer and Diederich, 2010, pp. 169–170)—also accommodates an account of increased subprocesses with intact subprocess-level processing rate. It too accords with empirical-performance patterns of task encoding demands, and diagnostic status^2^.

The expression of Sz encoding performance as an elevation in subprocesses with intact speed of subprocess execution is expected to extend to Stroop performance. The Stroop task is considered to be encoding intensive in its requirements for extracting task-prescribed properties from a stimulus complex, and more so when a color word and the ink in which it is written are incongruent. This condition demands the segregation of imperative from detracting stimulus features. If subprocess incrementation is the agent of increased latency, modeling Sz performance by releasing the subprocess parameter but fixing the subprocess-rate parameter (or their mixing distributions), across groups, should fit empirical performance data. Furthermore, diagnostic specifically of increased subprocesses, changes in the additivity of performance latency should be observed with increased encoding load (word-color incongruency) and the entry of Sz diagnostic status.

### Neuroconnectivity of Sz deviations in stimulus encoding

Abnormalities in fMRI-monitored neuro-activation during stimulus encoding have centered on the anterior cingulate cortex (ACC). Relative to controls, the pattern of activation in Sz has consisted of more diffuse, less ACC-channeled responding (Boksman et al., 2005; Neufeld et al., 2010; Ungar et al., 2010). Less ACC activation and more widespread co-activation at less ACC-proximal locations, has indicated diversion away from normal encoding-intensive sites. Encoding-rich tasks, or task segments, have involved the summoning of lexical associations to presented consonants (Word-Fluency task; Boksman et al., 2005); Stroop performance (Ungar et al., 2010); and encoding probe items, for memorial comparison to a set of memorized items (memory-search task; Neufeld et al., 2010).

The link between stimulus encoding and less ACC-centered neuro-activation has been relatively robust across Sz-groups—extending to first-episode, never-treated participants (Boksman et al., 2005). It furthermore has been associated with subtle dynamical differences in 7.0 Tesla measured ACC glutamatergic activity occurring to repeated blocks of Stroop-performance and rest periods (Taylor et al., 2015). For example, healthy controls have generated increased glutamate but not glutamine upon an initial block of randomized Stroop conditions, the opposite increase occurring for Sz participants.

## Modeling fMRI-monitored stroop performance

### Method

#### Overview

The Stroop task was performed during Blood Oxygenation Level Dependent (BOLD) neuro-imaging of functional activation at 7.0 Tesla for Sz, MDD, and HC participant groups. The Stroop paradigm combined selected conditions typically used in clinical studies (e.g., Perlstein et al., 1998), elaborated upon in light of Systems Factorial Technology (SFT; Townsend and Nozawa, 1995), as applied to analysis of Stroop performance (Eidels et al., 2010). Clinical science studies often include three conditions: the naming of the ink in which a word corresponding with the color is written (e.g., “green” written in green ink; *congruent* condition); the naming of the ink color in which a different-color word is written (e.g., “red” written in green; *incongruent* condition); and the reading of a non-color word, written in a color (e.g., “sheep” written in green; *neutral* condition).

Three conditions of the present paradigm required participants to name the color of the ink in which a color-word was written. In the congruent condition, the word and ink-color matched, and in the incongruent condition, they mismatched. In a third condition, the color of a color-patch, consisting of a row of 5 “x's,” was to be named (color only). A fourth condition (word only; red, green, blue, or yellow) required the reading of a word printed in white against a black background.

#### Participants

There were 16 participants in the Sz and each of the control (HC and MDD) groups who gave informed written consent according to local Ethics Board Approval Guidelines. Prospective volunteers with neurological or major medical illnesses, clinically significant head injury, other psychiatric disorders, MRI contra-indications, or substance abuse within the previous year were excluded from the study. Any healthy volunteer with a known family history of psychiatric disorder in a first or second degree relative was also excluded. A Stroop-task recording failure for one of the Sz participants reduced to 15 the number contributing to the cognitive-behavioral analysis for that group. The fMRI analyses, however, were applied to data from the original 16 Sz participants, on the reasonable assumption (supported below) that results from the remaining 15 participants nevertheless would generalize.

A consensus diagnosis was established on all participants by a psychiatrist and trained assistant with the Structured Clinical Interview for DSM-IV (First et al., 1997). Sz subjects were rated with the Scale for Assessment of Negative Symptoms and the Scale for the Assessment of Positive Symptoms (Andreasen, 1984a,b) and MDD patients were assessed with the Montgomery Asberg Depression Scale (Montgomery and Asberg, 1979) and the Young Mania Rating Scale (Young et al., 1978). Thirteen Sz patients were receiving atypical neuroleptics with Chlorpromazine Equivalent 426 ± 299 mg (2 taking olanzapine; quetiapine/venlafaxine; 2 taking risperidone; quetiapine/paliperidone/escitalopram; 4 taking paliperidone; clozapine; risperidone/escitalopram; quetiapine/escitalopram); and 2 patients were not medicated. Ten of the 16 MDD patients were receiving antidepressant medications at the time of the scan (bupropion/citalopram/ methylphenidate; venlafaxine; lamotrigine; desvenlafaxine; bupropion/citalopram; escitalopram; citalopram; sertraline; citalopram/mirtazapine/quetiapine; levothyroxine/melatonin). While it is possible that some of the medications will affect glutamatergic function, the actual functions of many of the above prescribed medications is unknown. Demographic information including age, handedness, education, parental education, clinical rating scores, and length of illness were collected in accordance with methods described in our previous study (Aoyama et al., 2011) and are shown in Table 1.

**Table 1:** **Participant demographics**.

**Group**	**Controls**	**MDD**	**SZ**	***p***
*n*	16	16	15	
Age	24.18 ± 4.67	22.62 ± 4.75	22.70 ± 2.98	0.510
M/F	10/6	5/11	12/3	**0.019**
R/L	14/2	14/2	15/0	0.842
Educ	3.06 ± 0.77	2.56 ± 0.63	2.20 ± 0.86	**0.010**
PEduc	3.13 ± 0.96	2.88 ± 0.81	3.20 ± 0.77	0.539
HAM-A		12.94 ± 10.86		
HAM-D		12.50 ± 9.11		
Mania		5.38 ± 6.79		
Montg		17.81 ± 10.68		
CPZ (mg)			368.83 ± 314.67	
SANS			9.60 ± 8.01	
SAPS			7.80 ± 10.67	
Illness duration (months)		28.56 ± 14.52	30.40 ± 15.86	

M/F, male/female; R/L, right/left; Educ, education rating of the participant (1, gr. 10 or lower; 2, completed high school; 3, 1–3 years of college/university; 4, >3 years of college/university); PEduc, education rating of the participant's parent (1, gr.10 or lower; 2, completed high school; 3, 1–3 years of college/university; 4, >3 years of college/university); H Anx, Hamilton Anxiety Scale; Hamilton, 1959; H Dep, Hamilton Depression Scale; Hamilton, 1960; Mania, mania rating from the Young Mania Rating Scale; Young et al., 1978; Montg, result of the Montgomery Asperg Depression Scale; Montgomery and Asberg, 1979; CPZ, chlorpromazine equivalent; SANS, Scale for Assessment of Negative Symptoms; Andreasen, 1984a; SAPS, Scale for Assessment of Positive Symptoms; Andreasen, 1984b; p, ANOVA test for significance (alpha = 0.05, two-tailed), bold values indicate significance.

### Procedure

#### Stroop task

The stimuli (described above) were presented for 2 s and the subjects were asked to respond as quickly and accurately as possible within this time frame. A trial began with 1 s of cross fixation (“+”) in the center of the screen. All visuals were presented with a black background. Every participant practiced outside the scanner until they achieved 80% correct responses. Participants then underwent an MRI protocol that consisted of a total of 8 min of the Stroop Task activity for the purpose of examining glutamatergic activity in the ACC (Taylor et al., 2015). The participants were then removed from the MRI scanner for a 30 min break before re-entering to complete the fMRI component (providing the current functional connectivity analysis), which consisted of nine, 1-min blocks whose trials cycled between cross fixation and Stroop-Task presentations, for a total of 4 min of Stroop activation during the fMRI. A total of 80 Stroop stimuli were presented, twenty from each condition (i.e., congruent, incongruent, word-only, and color-only) presented pseudo-randomly throughout the session (consistent order between participants). The paradigm was written and presented using PsychoPy (Peirce, 2007).

#### MRI signal acquisition and pre-processing

All data was acquired on a 7.0 Tesla Agilent/Magnex head-only MRI (Agilent, Inc., Walnut Creek, California, USA) with a Siemens AC84 head gradient coil (Siemens, Erlangen, Germany), located at the Center for Functional and Metabolic Mapping at the University of Western Ontario's Robarts Research Institute. A transmit-only/receive-only head coil with 15 transmitters and 23 receivers and a built in mirror was used for all scans (Gilbert et al., 2011). A transmit-field shimming approach facilitated optimized homogeneity of the transmit field for each scan (Curtis et al., 2012). The magnetic field uniformity was adjusted automatically using RASTAMAP (Klassen and Menon, 2004). The fMRI volumes were localized using anatomical MRI images acquired with fast low-angle shot 2D (FLASH2D) images [5 slices, repetition time (TR) = 6.3 ms, echo time (TE) = 3.5 ms, flip-angle = 11°, gap between slices = 1 mm, thickness = 2 mm, field-of-view = 30 × 30 cm, matrix size = 128 × 128] in each of the sagittal, transverse, and coronal orientations. The fMRI images were then acquired using an echo-planar imaging (EPI) sequence (45 slices, interleaved sliced order, repetition time (TR) = 3 s, echo time (TE) = 18 ms, flip-angle = 90°, gap between slices = 0.2 mm, thickness = 2 mm, field-of-view = 22 × 22 cm, matrix size = 110 × 110, GRAPPA = 3, 4 steady state scans), angled to the AP line and aligned with the top of the brain.

Functional and anatomical data were preprocessed using Statistical Parametric Mapping 8 (SPM8, Wellcome Trust Centre for Neuroimaging, London, UK) implemented in MATLAB R2013a (Mathworks Inc., Sherborn, MA, USA). Individual functional images were corrected for motion by realignment to the first volume of the session. All images were spatially normalized (2 × 2 × 2 mm) to an EPI template in MNI space and spatially smoothed with a 6 mm full width at half maximum (FWHM) isotropic Gaussian kernel.

#### Connectivity estimation and statistical criteria

Statistical analysis proceeded in four steps. In a first-level analysis, pre-processed fMRI data from each individual participant were entered in a voxel-wise general linear model with design matrices derived from the individualized instance of the Stroop paradigm (i.e., epoch-related regressors). In the second step of this first-level analysis, participant data obtained in the first step was entered in a full-factorial design with a between-participant factor of group (3 levels: HC, Sz, MDD) and a within-participant factor of stimulus encoding load (2 levels: Low, High; stipulated below). This step was used to identify brain regions activated during transaction of the Stroop task common to all groups and all stimulus encoding loads. In a third step, a second-level analysis used the maximally-activated clusters obtained in the previous step as seed regions of a functional connectivity analysis. Specifically, we identified regions with significant psychophysiological interactions (PPI) with the seed regions (Friston et al., 1997). The seed region's activity time-course was obtained by spatially averaging the activity of all imaging voxels within a sphere of 10 mm radius centered on the coordinates of the maximally activated voxel within the selected cluster at each time point. The results of this analysis represent regions whose BOLD fMRI time course of activity significantly covaried with the activity of the seed region during performance of the Stroop task, relative to the baseline conditions (cross fixation). These regions are said to be functionally connected to the seed region for the explicit purpose of Stroop task transaction.

Note that, for the purpose of the fMRI analysis, two stimulus encoding conditions—low and high—were constructed as follows. The high encoding load consisted of the color-word incongruent condition, for all groups. The low encoding load for the HC and MDD groups comprised an amalgamation of the color-word congruent and color-only conditions, and that for the Sz group comprised an amalgamation of the color-only and word-only conditions (elaborated upon below; Section Data Properties Narrowing Model Selection). In a final step, the results of the PPI analysis were entered in a second-level full factorial design with group as a between subject factor and encoding load as a within-subject factor, to identify brain regions connected to the seed region in a group- and load-specific manner.

To distinguish common protuberant regions of functional activation (second step of our analysis), we adopted a whole-brain family-wise error (FWE) rate of 0.05. To balance Type-I error protection against false negatives in our functional-connectivity analysis (third step, above), our SPM t-maps were thresholded at *p* < 0.001 (voxel-level) with minimal cluster size *k* = 10 (Lieberman and Cunningham, 2009; see also, Ahn et al., 2011). Our SPM8 analysis used a within-cell error term (*df* = 90) throughout, with its potential increase in Type-I error protection for all contrasts involving the High-Low encoding-load factor (Kirk, 2013, chapters 10, 12).

#### Cognitive-behavioral data organization and analytical methods

Means and inter-trial variances for responses within the 2 s trial-time window were computed and adjusted for movement time. A value of 0.160 s was subtracted from the means, and (0.036 s)^2^ was subtracted from inter-trial variances (Woodworth and Schlossberg, 1954; Townsend, 1984; cf. endnote 4 of Townsend and Wenger, 2004).

Because trial numbers had to accommodate reasonable demands on clinical participants performing in an MRI environment, it was necessary to aggregate data across participants, while avoiding the conflation of systematic individual differences (Estes, 1956; Neufeld and Gardner, 1990). As previously done in clinical cognitive science, significant heterogeneity could be accommodated through mixture-model structures, allowing for inter-participant differences in model properties (e.g., Batchelder, 1998, 2007; Riefer et al., 2002). With relative homogeneity of performance, on the other hand, a group could be represented as a homogeneous participant according to the data centroid (e.g., Townsend, 1984; Carter and Neufeld, 1999; Neufeld et al., 2007b).

Tacks to modeling comprised a combination of parametric and nonparametric methods. Estimation of mixture-model hyper-parameters and tests of empirical fit for the current candidate architectures have been described elsewhere (Neufeld et al., 2007b, 2010). Alternatively, given contraindication of systematic individual differences, parameter estimates for candidate architectures were directly available through the method of moments (moment matching; e.g., Evans et al., 2000). Numerical simulation indicated that moment-matching estimates equalled those of maximum likelihood, within a constant of proportionality. Estimates also agreed with those from direct solutions, where manageable subsets of predictions were equated to corresponding empirical values, followed by solving simultaneously (moment fitting).

Testing of model predictions against observed latencies, and mean inter-trial variances, used the following ANOVA-based χ^2^ formats (Snodgrass and Townsend, 1980; Carter and Neufeld, 1999; see, Kirk, 2013). The first was

(1) $$\begin{array}{l} \chi^{2}=\sum_{w=1}^{W}\frac{{\left(\chi_{{observed}_{w}}-\mu_{model-{predicted}_{w}}\right)}^{2}}{\sigma_{{model-predicted}_{w}}^{2}} \end{array}$$

with *df* = *W* − (*number of parameter estimates*), where *W* is the total number of combinations of groups and performance conditions contributing to model-predicted empirical values. Here, the term χ_*observed*_*w*__ is either the *w*th empirical-sample mean latency, or inter-trial-latency variance; μ_*model*−*predicted*_*w*__ is the corresponding modeled mean, or inter-trial variance; and $\sigma_{{model-predicted}_{w}}^{2}$ is the model-prescribed variance of the sample mean or inter-trial variance. In the case of mean latency, $\sigma_{{model-predicted}_{w}}^{2}$ becomes (*model-predicted inter-trial variance*_*w*_)/*q*_*w*_, where *q*_*w*_ is the number of task trials making up χ_*observed*_*w*__. Where χ_*observed*_*w*__ consists of inter-trial variance, $\sigma_{{model-predicted}_{w}}^{2}$ becomes $\frac{2{\left(\sigma_{model-predicted_{w}}^{2}\right)}^{2}}{q_{w}}$. Division is by *q*_*w*_ rather than *q*_*w*_ -1 because maximum-likelihood estimates were used for sample estimates (e.g., Evans et al., 2000). It is assumed that sample values are normally distributed, which is defensible in view of the Central Limit Theorem.

A second version of χ^2^ was

(2) $$\begin{array}{rc} \chi^{2} & =\overset{W}{\underset{w=1}{\sum }}\frac{q_{w}{\left(Mean_{latency_{observed_{w}}}-\mu_{model-predicted_{w}}\right)}^{2}}{\sigma_{model-predicted_{w}}^{2}}\\ +\overset{W}{\underset{w=1}{\sum }}\overset{q_{w}}{\underset{i=1}{\sum }}\frac{{\left(\chi_{iw_{observed}}-Mean_{latency_{observed_{w}}}\right)}^{2}}{\sigma_{model-predicted_{w}}^{2}} \end{array}$$

with $df\;=\;\sum_{w\;=\;1}^{W}q_{w}-\left(number\;of\;parameter\;estimates\right)$. Here, the individual latencies χ_*iw*_*observed*__ in the double summation are less likely to be normally distributed. In actual testing, both Equations (1) and (2) nevertheless had to agree on tenability of model fit. Essentially, Equations (1) and (2) address the degree to which the proposed model specifies a population whose summary statistics are coherent with empirical values. Results from these equations also agreed with those from selected applications of multinomial-likelihood *G*^2^(≈ χ^2^), and Pearson χ^2^ applied to proportions of responses binned into 5 equally-spaced latency intervals (exemplified below).

### Results

#### Participant characteristics

Significant differences occurred with respect to participant education, but not parental education (parent with the higher education-level; Table 1). Although the proportion of males in neither the Sz nor the MDD group differed significantly from that of the HC group, *p* > 0.10, the two patient groups differed significantly from each other, $\chi_{\left(1\right)}^{2}=7.429$, *p* = 0.0064. Overall, however, males responded significantly faster than females, *t*_(141)_ = 3.8862, *p* < 0.001, partial η^2^ = 0.269. Any effect of sex differences on response latency therefore would be in the direction of increased speed in the Sz group. Note, as well, that individuals with Sz, especially those with paranoid symptomatology (all but 3 of the present sample) tend more often to be male. The ratio of female to male prevalence rates for MDD, in turn is 1.64 (Romans et al., 2007). Eliminating such (intrinsic) group differences risks the introduction of other, more intractable issues of interpretation (e.g., Cochran, 1957; Evans and Anastasio, 1968; Meehl, 1971).

There were no significant correlations between Chlorpromazine (CPZ) daily-dosage equivalents (mg. per day) and any of the performance variables (e.g., *r*_*CPZ, all response latencies*_ = −0.179, *p* = 0.522).

#### Data overview

Results from analyses of all latency responses, and those from analyses of correct-only responses were highly similar throughout, and analyses of correct-only responses yielded no additional information (proportion correct generally exceeded 0.90, and in no case were there significant group differences on proportion correct). Therefore, only results based on all responses are reported (cf. Link, 1982). Table 2 presents adjusted latency means and inter-trial variances, in each case along with corresponding inter-participant standard deviations—taking into account all responses occurring inside the 2-s trial intervals. Also listed are the percent of correct trials and their inter-participant standard deviations. Table 3 presents latency means and inter-trial variances, along with inter-participant standard deviations, but for correct responses only.

**Table 2:** **Response time latencies to each of the Stroop conditions with inter-participant standard deviations using all responses**.

**Group**	**Condition**	**Means (s)**	**Variances (s)^a^**	**% Correct^b^**	**% Correct^c^**
HC	Congruent	0.6072 ± 0.0922	0.0479 ± 0.0205	0.9500 ± 0.1125	0.9767 ± 0.0372
	Incongruent	0.7885 ± 0.1714	0.0784 ± 0.0242	0.8875 ± 0.1147	0.9100 ± 0.0737
	Color-only	0.6118 ± 0.1096	0.0381 ± 0.0334	0.9563 ± 0.1276	0.9733 ± 0.0417
	Word-only	0.7081 ± 0.0840	0.0614 ± 0.0160	0.9438 ± 0.1250	0.9867 ± 0.0399
MDD	Congruent	0.6362 ± 0.0866	0.0531 ± 0.0325	0.9906 ± 0.0202	
	Incongruent	0.8854 ± 0.1088	0.0621 ± 0.0227	0.9625 ± 0.0532	
	Color-only	0.6495 ± 0.0787	0.0492 ± 0.0236	0.9781 ± 0.0364	
	Word-only	0.7111 ± 0.0943	0.0539 ± 0.0241	0.9781 ± 0.0364	
SZ	Congruent	0.6645 ± 0.1029	0.0791 ± 0.0519	0.9633 ± 0.0352	
	Incongruent	0.8906 ± 0.1245	0.0764 ± 0.0336	0.8700 ± 0.1709	
	Color-only	0.7075 ± 0.1267	0.0756 ± 0.0378	0.9567 ± 0.0417	
	Word-only	0.7060 ± 0.1208	0.0613 ± 0.0355	0.9633 ± 0.0516	

HC, healthy controls; MDD, Major Depressive Disorder; SZ, schizophrenia.

a Variances: inter-trial latencies.

b Non-responses are considered incorrect.

c Excluding responses from one healthy control subject who confused green and yellow buttons during the fMRI task.

**Table 3:** **Response time latencies to each of the Stroop conditions with inter-participant standard deviations using correct responses only**.

**Group**	**Condition**	**Means (s)**	**Variances (s)^a^**
HC	Congruent	0.5988 ± 0.0964	0.0458 ± 0.0210
	Incongruent	0.7893 ± 0.1759	0.0772 ± 0.0244
	Color-only	0.6075 ± 0.0879	0.0367 ± 0.0155
	Word-only	0.7051 ± 0.1117	0.0621 ± 0.0345
MDD	Congruent	0.6331 ± 0.0839	0.0492 ± 0.0277
	Incongruent	0.8870 ± 0.1090	0.0616 ± 0.0224
	Color-only	0.6479 ± 0.0929	0.0487 ± 0.0244
	Word-only	0.7109 ± 0.0785	0.0544 ± 0.0235
SZ	Congruent	0.6545 ± 0.0987	0.0734 ± 0.0519
	Incongruent	0.8914 ± 0.1277	0.0749 ± 0.0331
	Color-only	0.6947 ± 0.1231	0.0665 ± 0.0312
	Word-only	0.6975 ± 0.1121	0.0563 ± 0.0313

HC, healthy controls; MDD, Major Depressive Disorder; SZ, schizophrenia.

a Variances: inter-trial latencies.

Note that mean latencies reported in Tables 2, 3 were computed directly from all values, rather than as the average of participant-wise means. Data ensembles from individual participants therefore *de facto* were weighted according to their numbers of valid observations.

Likewise, inter-trial variances were computed as the sum of squared deviations from the grand mean, above, of all observations in the group-condition combination, divided by the total number in that combination (maximum-likelihood estimate; e.g., Evans et al., 2000). Like mean latency values, individual participants' data ensembles were consequently weighted according to their numbers of valid observations. Although such variance estimates included between-participant variance in mean latencies, they were logically coherent with a homogeneous-participant approach to data treatment (further elaborated upon, below; Section Within-Group Performance Homogeneity), including relative homogeneity of mean latencies.

One HC participant reported having accidentally reversed green and yellow response buttons during the experimental trials (despite meeting the 80% correct criterion on practice trials). Values for percent correct, and their standard deviations, with this participant excluded, are presented in the last two columns of Table 2. It was decided to retain this individual's latency data throughout, because apart from the response-button reversal, Stroop-item processing of principle interest was deemed to have occurred. Again, results from analyses of all responses within the 2-s trial time interval, and those from correct responses only, essentially were interchangeable.

#### Within-group performance homogeneity

It was first examined as to whether mean latencies and inter-trial variances, across participants within groups, tenably emanated from a single population, according to conformity to an hypothesized normal distribution. To this end, Kolmogorov-Smirnov (Lillifors corrected) and Shapiro-Wilks tests were applied (as done, e.g., in Neufeld et al., 2010). None of these 24 tests—4 Stroop conditions per group, on each of the above quantities—were significant, despite appreciable statistical power (Wilcox, 1997).

With performance data tenably emanating from a single population per group, questions remain regarding relative homogeneity of inter-participant performance within each group. This possibility was initially examined according to coefficients of variation (*c* of *v*). Results generally did not indicate any over-dispersion that would signal systematic individual differences in model operation (see, e.g., Batchelder and Riefer, 2007). For example, the *c* of *v* calculated on the inter-participant standard deviation, pooled across conditions and groups, divided by the grand mean was 0.1518. This value was significantly lower than that of a provisional benchmark of 0.297 for mixture-model status of response latencies (Neufeld et al., 2010; McKay's approximate $\chi_{\left(46\right)}^{2}=12.7966$, *p* → 1.0). A further example is the *c of v* for Sz participants, under the incongruent condition of 0.139, McKay's approximate $\chi_{\left(14\right)}^{2}=3.27$, *p* = 0.9977.

An additional assessment of performance data, for evidence of inter-participant heterogeneity in model operation, appropriated a version of coefficient alpha that addressed homogeneity of response proportions over the 5 bins of adjusted-latency intervals (2 s divided into 0.4 s segments). This version of coefficient alpha was 1 − (*Mean-square*_*participants* × *bins*_/*Mean-square*_*bins*_) (e.g., Neufeld and McCarty, 1994). Homogeneity of values was supported according to an overall mean value, taken across conditions and groups, of 0.985. Similar support was obtained according to computations of the proportion of variance accounted for by the bins in each group's bins × participant layout (cf. Schmitt, 1996). Note that a χ^2^ test on the bin-by-participant frequencies was contraindicated by sparseness of some individuals' cell frequencies (Delucchi, 1993; Tollenaar and Mooijaart, 2003). Altogether, these preliminary analyses indicated that group centroids were not unrepresentative of data configurations for the separate participants.

#### Data properties narrowing model selection

Given the uniform temporal properties of responding, attention is turned to the stochastic cognitive modeling of performance and its accounting for differences across groups. With a tenable stochastic model in hand, cognitive-process dynamics are poised for projection onto those of neuro-connectivity monitored during task trials.

Examination of the pattern of latencies in Table 2 discloses marked similarities and differences in values across groups and conditions. Mean latencies for the color-only and congruent conditions were highly similar, for both the HC and MDD groups (2-tailed *p*'s ≥ 0.455). In the case of the Sz group, those for the color-only and word-only conditions were nearly identical (2-tailed *p* = 0.981).

Also apparent was the similarity in differences between the color-only (≈congruent) and incongruent condition means for the HC group, and that between the color-only (≈word-only) and incongruent means for the Sz group. When the HC color-only and congruent means were amalgamated, and the Sz color-only and word-only means were amalgamated, the group difference in the contrast for the incongruent condition (i.e., the 2nd-order difference) was only 0.00633, *t*_(*Huynh-Feldt-epsilon-corrected df* = 124)_ = 0.24011, *p* = 0.798, 2-tailed. Therefore, although these groups differed significantly from each other in their latency values (e.g., 2-tailed *p* = 0.039 and 0.012 for the incongruent and color-only conditions), and differences between the incongruent and other conditions were highly significant throughout (2-tailed *p* = 0.003), the effects on latency of HC-Sz group status, and higher (incongruent condition)- vs. lower-encoding load (color only ≈ congruent _*HC*_; color only ≈ word only _*Sz*_), were highly additive. Such additivity shrinks considerably the set of eligible model structures and parameter changes across groups.

The equivalent latency of color-only and word-only conditions was unique to the Sz group. Note that this result was not observed in the earlier session on fMRS, where 40 trials had been performed under each Stroop condition (Taylor et al., 2015). The present equivalence therefore was specific to the Sz group in the second session of task performance.

Also unique to the Sz group was a seeming reduction in latency under the congruent condition, relative to the color-only (≈word-only) condition. A more pronounced congruent-condition facilitation effect for Sz participants has been previously indicated in the literature on their Stroop performance (e.g., Perlstein et al., 1998). In SFT terms, “target-redundancy gain” under the congruent condition may have specially benefited the Sz group. Stochastic mechanisms endowing redundancy gain include “statistical advantage,” occurring to an independent parallel-channel architecture (see e.g., Townsend et al., 2007; Khodadadi and Townsend, 2015); transition to a highly efficient capacity-workload architecture, (notably a co-active parallel-channel structure, e.g., Townsend et al., 2007); or a structure with cross-channel facilitation (e.g., Johnson et al., 2010, their Equation A.1.1, Box II); or possibly transition to a Gestalt- model structure (Townsend and Ashby, 1983, chapter 13; Snodgrass and Townsend, 1980).

Certain inter-condition differences in latency means were in keeping with such possibilities. Specifically, unlike the HC group, with little evidence of redundancy gain, the Sz group displayed significantly lower latencies under the congruent condition than under their color-only≈word-only conditions (2-tailed *p* = 0.037). The second-order difference (*mean*_*color-only—word-only amalgam*_ − *mean*_*congruent*_)_*Sz*_ − (*mean*_*color only*_ − *mean*_*congruent*_)_*HC*_, however, fell short of significance, *t*_(124)_ = 1.3413, *p* = 0.0911, one-tailed. This marginal evidence from mean latencies for Sz-specific congruency facilitation nevertheless is formally probed (below), in terms of estimated differences in the presence of redundancy-gain mechanisms.

Also evident in Table 2 is a more pronounced color-only—incongruent latency difference for the MDD group (0.24 s) than for the HC or Sz groups (0.18 s). The test on the second-order difference, (*mean*_*color-only—congruent amalgam*_ − *mean*_*incongruent*_)_*MDD*_ − (*mean*_*color-only—congruent amalgam*_ − *mean*_*incongruent*_)_*HC*_, *t*_(*Huynh-Feldt corrected df* = 124)_ = 2.486, *p* = 0.0143, *dz* = 0.507, signaled a possible disproportionate MDD cognitive workload-capacity reduction. Across-channel (color-word) impedance may have occurred in the MDD processing system operative during their incongruent trials. The MDD group's performance data is also evaluated, expressly for this possibility, below.

### Candidate model structures

#### Mean diagnostics

Focusing initially on the Sz and HC groups, the preliminary analysis on latency data, above, was mean diagnostic of eligible models. Candidate model structures and parameter changes were required to generate additive effects on mean latencies of elevation in encoding load—transition from color-only, and its congruent or word-only equivalent, to the incongruent condition—and Sz diagnostic status. This additivity corresponds to the associated near-zero second-order difference (0.00633), above.

A model generating mean additivity with increased encoding load and Sz clinical diagnosis is the Erlang distribution, above. With mean *k*′/*v, k*′ being the number of constituent processing operations (subprocesses), and *v* their rate of dispatch (subprocess-level capacity), elevation in *k*′ across encoding load and Sz diagnosis produces the requisite mean additivity.

Other structures (reviewed in Neufeld et al., 2010) also produce the requisite configuration of latency means. One such structure is the Independent Parallel Moderately Limited Capacity structure (IPMLC; Townsend and Ashby, 1983), whose *E*(*T*) is

$$\begin{array}{l} E{\left(T\right)}_{IPMLC}=\sum_{i=1}^{k\prime }\frac{1}{\left(k\prime -i+1\right)\left(\frac{v}{k\prime }\sum_{j=1}^{k\prime }\frac{1}{j}\right)} \end{array}$$

Another, is a parallel structure with unlimited capacity during completion of the first subprocess, followed by a decline and then partial recovery—known as the First-Stage Unlimited Capacity Parallel model (FSUCP; Neufeld et al., 2010; originating with Townsend, 1984). For this model, *E*(*T*) is

$$\begin{array}{l} {E\left(T\right)}_{FSUCP}\;=\;\sum_{i=1}^{k\prime }\frac{1}{\left(k\prime -i+1\right)\left(\frac{k\prime v}{2\left(i-\frac{1}{2}\right)\left(k\prime -1+1\right)}\right)}. \end{array}$$

These expectations emanate from density functions *f* (*t*) that are instances of the General Gamma distribution (McGill and Gibbon, 1965). The density function of the General Gamma distribution is

$$\begin{array}{l} {f\left(t\right)}_{General\;Gamma}\;=\;\overset{k\prime }{\underset{i\;=1}{\sum }}C_{ik\prime }c_{i}e^{-c_{i}t} \end{array}$$

where

$$\begin{array}{l} C_{ik\prime }=\frac{1}{\overset{k\prime }{\underset{j=1;j\neq i}{\prod }}\left(1-\frac{c_{i}}{c_{j}}\right)} \end{array}$$

and *c*_*i*_ is the exponential–distribution rate parameter for the *i*th stage of processing, *i* = 1.2, …*k*′. For the IPMLC model, *c*_*i*_ is

$$\begin{array}{l} c_{i_{IPLMC}}=\left(k\prime -i+1\right)\frac{v}{k\prime }\sum_{j=1}^{k\prime }\frac{1}{j}, \end{array}$$

and for the FSUC model, *c*_*i*_ is

$$\begin{array}{l} c_{i_{FSUC}}=\frac{\left(k\prime -i+1\right)k\prime v}{2\left(i-\frac{1}{2}\right)\left(k\prime -i+1\right)}. \end{array}$$

Each of the above candidates is considered further in the next section.

#### Inter-trial variance considerations

Like latency means, inter-trial variances in principle can contribute to the selection of eligible model structures and their parameter changes across experimental factors. For example, inter-trial variance of the Erlang distribution is *k*′/*v*^2^, which, again, increases linearly with an increase in *k*′. The variance of the FSUCP model is

$$\begin{array}{l} Var{\left(T\right)}_{FSUCP}=\sum_{i=1}^{k^{\prime }}\frac{1}{{\left[(k^{\prime }-i+1)(\frac{k^{\prime }v}{2\left(i-\frac{1}{2}\right)\left(k^{\prime }-i+1\right)})\right]}^{2}}\\ \;=\frac{1}{3}\frac{\left(4{k^{\prime }}^{2}-1\right)}{k^{\prime }v^{2}}, \end{array}$$

which to all intents and purposes is also linear on *k*′. That for the IPMLC model is

$$\begin{array}{l} {Var\left(T\right)}_{IPMLC}=\sum_{i=1}^{k\prime }\frac{1}{{\left[\frac{\left(k\prime -i+1\right)v}{k\prime }\left(\sum_{j=1}^{k\prime }\frac{1}{j}\right)\right]}^{2}}, \end{array}$$

which accelerates on *k*′.

A difficulty with the diagnostic significance of inter-trial variances, and those of higher-order moments, is the instability of their empirical estimates [e.g., Ratcliff, 1979; consider the model-predicted variances of empirical means vs. those of inter-trial variances of Equation (1), above].

To illustrate, using the mean and inter-participant standard deviation averaged across all groups and conditions, the *c* of *v* for the mean latency (0.1585) was almost ¼ that of the variances (0.5935). Likewise, taken as a representative surrogate for population values the mean and inter-trial variance of the HC group under the incongruent condition, the *c* of *v* for the sample mean latencies (0.081479) once again was roughly ¼ that of the variances (0.32444).

With these qualifications in mind, sample variances somewhat decelerated opposite to theoretical variances of the IPMLC model when supposing a progressive increase in *k*′ with greater encoding load and Sz group membership. The linear increases in variances of the Erlang and FSUCP structures at minimum were not opposite to the sample values' second-order difference for encoding-condition and diagnostic groups.

The empirical variances' relative instability presents a challenge to models in which variances figure into parameter estimation and tests of empirical fit. On balance, the Erlang distribution was selected as the structure to be tested for empirical fit given the random departure of sample variances from model linearity on *k*′, and considering its straightforward composition. Although this selection was spawned by the Sz and HC groups' performance data, in the interests of parsimony, it was also tested against data of the MDD group.

#### Hazard-function considerations

Note that the Erlang distribution's hazard function *h*(*t*) = *f* (*t*)/*S*(*t*), *f* (*t*) being the density function and *S*(*t*) the survivor function, increases monotonically over *t*. Here, the density function is that stated in Section Mathematically Modeled Cognitive Deviation in Sz, and the survivor function is

$$\begin{array}{l} S{\left(t\right)}_{Erlang}\;=\;1-\overset{\infty }{\underset{j=k\prime }{\sum }}\frac{{\left(vt\right)}^{j}}{j!}e^{-vt}=\overset{k\prime -1}{\underset{j=0}{\sum }}\frac{{\left(vt\right)}^{j}}{j!}e^{-vt}=\frac{\Gamma \left(k\prime ,vt\right)}{\Gamma \left(k\prime \right)} \end{array}$$

Where Γ(*k*′, *vt*) is the incomplete gamma function $\int_{vt}^{\infty }x^{k\prime -1}e^{-x}dx$. Empirical estimates of *h*(*t*), however, have been non-monotonic, first increasing, then decreasing over *t* (Bloxom, 1984, 1985; Luce, 1986).

Note that latency aggregates, such as those emphasized in the current work, nevertheless can be functions of *h*(*t*), with values of these functions themselves being similar for monotonic and non-monotonic shapes. For example, *S*(*t*), a function that bears directly on the binning of proportions of empirical latencies according to intervals of *t*, can be expressed for any continuous distribution as

$$\begin{array}{l} S\left(t\right)=e^{-\int_{0}^{t}h\left(t^{\prime }\right)dt^{\prime }} \end{array}$$

Simulation of *S*(*t*)'s from monotonic and non-monotonic *h*(*t*)'s—the latter created via probability mixtures (Barlow and Proschan, 1975)—shows that their trajectories across *t* can essentially converge.

Observe, as well, that the *n*th moment *E*(*T*^*n*^) is equal to

$$\begin{array}{l} E\left(T^{n}\right)=n\int_{0}^{\infty }S\left(t\right)t^{n-1}dt. \end{array}$$

Inferences from *S*(*t*) and *E*(*T*^*n*^) generated by monotonic *h*(*t*)'s, as opposed to certain non-monotonic extensions, therefore, are arguably not imperiled given acceptable model fit at this level of analysis.

### Parameter estimation and tests of empirical fit

Results of parameter estimation and tests of empirical fit were similar for the Sz and HC groups whether considered separately or alongside the MDD group. For brevity, therefore, we report those for all three groups taken together.

Parameter estimation commenced with moment-matching solutions for the subprocess-rate parameter, *v*, separately at each combination of group and encoding-load condition (e.g., Evans et al., 2000). For all three groups, higher encoding-load was represented by the incongruent condition; again, the color-only and congruent conditions were amalgamated as low-encoding load for the HC and MDD groups, and likewise the color-only and word-only conditions for the Sz group. These estimates of *v* were then simply averaged, with the resulting value provisionally fixed for all groups. Subprocess number *k*′ was estimated using the mean and inter-trial variance, for each group and encoding load. The estimates were then, in turn, averaged at each group-encoding-load combination, and fixed at those combinations. The parameter *v* was subsequently re-estimated at each of these combinations, with its overall mean in turn being fixed for all groups. As estimates essentially converged at this point, they were retained for subsequent tests of empirical fit.

Parameter estimates were 12.0698 for *v*. The estimate of *k*′ for the HC group was 7. This value was incremented for all three groups under the incongruent condition by an estimated constant *h* = 3. The added subprocess-number for the Sz group was *g* = 2, and that for the MDD group was *g*′ = 1. Estimated subprocesses thus ranged from *k*′ = 7 to *k*′+*h* + *g* = 12.

**Figure 1:**
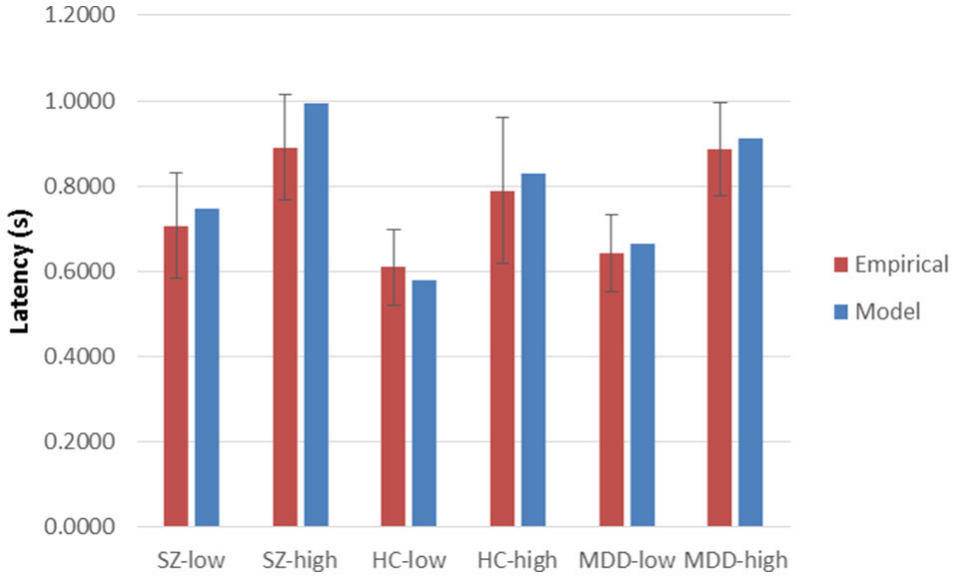
**Empirical latencies and model predictions, across High and Low Encoding Loads and diagnostic groups**. Sz, Schizophrenia participants; HC, Healthy Controls; MDD, Major Depressive Disorder participants. Low Encoding Load: Mean of color-only and congruent Stroop conditions, for MDD and HC groups; Mean of color-only and word-only conditions for Sz group. High Encoding Load: Incongruent color-word condition for all groups. Error bars are standard deviations (pooled for low-encoding-load conditions) across participants within groups and Encoding Load.

**Figure 2:**
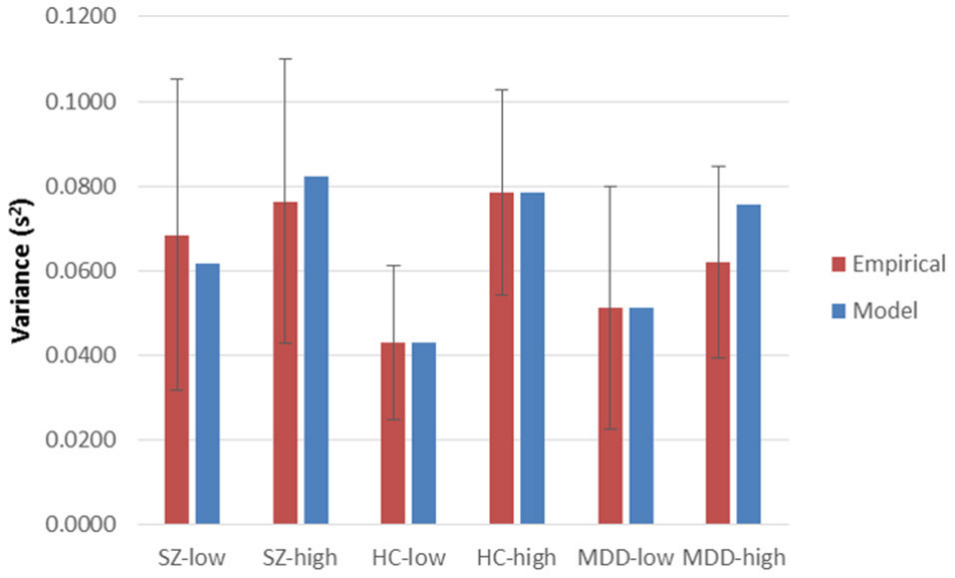
**Empirical inter-trial variances in latencies and their model predictions, across High and Low Encoding Loads and diagnostic groups**. Sz, Schizophrenia participants; HC, Healthy Controls; MDD, Major Depressive Disorder participants. High and Low Encoding Loads, and error bars, are as those for mean latencies (Figure 1).

Using Equation (1), $\chi_{\left(7\right)}^{2}$ was 6.26457, *p* = 0.5092. Similarly, with Equation (2), $\chi_{\left(174\right)}^{2}$ was 179.038, *p* = 0.3740. Probability values of Equation (2), computed separately at each combination of group and encoding load, ranged from 0.1789 (HC, incongruent) to 0.5482 (MDD incongruent), with a mean of 0.3700. Plots of empirical latencies and inter-trial variances, as set against their model predictions, are presented in Figures 1, 2. These results are called upon when integrating the dynamics of Stroop-performance with those of the fMRI hemodynamic response function (hrf), below.

### Formal analysis of possible Sz word-color congruency facilitation

The possible Sz-specific performance facilitation under the congruent word-color condition was examined using a combination of parametric and distribution-general (SFT) strategies. Mechanisms of stochastic-model structures that are potentially responsible for congruency facilitation are those of redundancy gain, occurring when multiple targets are co-present on a given trial. Both the word and color can be considered candidate stimulus features to the extent that reporting either one is an eligible response, as is the case for their solo appearance. In this way, the congruent condition potentially qualifies as a double target relative to the word-only and color-only conditions^3^. Candidate mechanisms of redundancy gain include the following: statistical advantage accompanying the operation of an independent (regular) parallel model, with unlimited processing-channel capacity (UCIP model); transition to a highly efficient, super workload-capacity structure, (notably represented by co-active, or cross-channel facilitative parallel structures); and transition to a Gestalt-model structure. Each of these possibilities now is taken up in turn.

#### Statistical advantage, super-capacity, and related model structures

Statistical advantage occurs in a redundant-target condition when two independent target-processing channels sum their workload capacity together toward attaining a single sufficient completion. Statistical advantage can be quantified as the sum of the individual channels' integrated hazard functions

$$\begin{array}{ll} \int_{0}^{t}h\left(t^{\prime }\right)dt_{color}^{\prime }\;+\;\int_{0}^{t}h\left(t^{\prime }\right)dt_{word}^{\prime }\\ = & \;-\text{ln}{\left(S\left(t\right)\right)}_{color}\;-\text{ ln}{\left(S\left(t\right)\right)}_{word}, \end{array}$$

in view of the equality $S\left(t\right)\;=\;e^{-\int_{0}^{t}h\left(t^{\prime }\right)dt^{\prime }}$. This sum is also expressed as, “the *Capacity Index* for color, added to the *Capacity Index* for word,” denoted *CI*_*color*_ + *CI*_*word*._ The *Capacity Index* for the double target condition, in turn, is denoted *CI*_*color*,_ _*word*_.

Capacity properties of the processing system can be assessed according to the SFT's Capacity–OR-Coefficient,

$$C_{OR}T\;\equiv \;\frac{CI_{color,word}}{CI_{color}+CI_{word}},$$

(Townsend and Nozawa, 1995).

Statistical advantage, a property of the UCIP model structure, is identified as a *C*_*OR*_*T* value of 1.0. In line with the expressions, above, this value indicates that channel processing-capacity is unaffected when channels are added to the processing system. Values of *C*_*OR*_*T* exceeding 1.0 imply system super-capacity, and those less than 1.0 signify limited capacity. Super-capacity occurs when channel-level processing speed actually increases with additional channels, or when a highly efficient system architecture is operative. In contrast, limited capacity occurs when channel-level speed diminishes with additional channels. As a special case, capacity is said to be “fixed” if single-target values are spread across target-processing channels in a redundant-target condition. The *C*_*OR*_*T* of a fixed, limited-channel system is 0.5.

As a possible agent of congruency facilitation, super-capacity can take place with highly efficient processing structures. In the case of a co-active parallel structure applied to redundant targets, the signals (aligned, e.g., with subprocess completions) of each independent processing channel are pooled with those of the other channel—as tributaries to a common conduit—with the trial being finalized when the required complement is reached. A cross-channel facilitative super-capacity system, on the other hand, terminates when completions of one or the other target-processing channel reaches a criterial level. The channels in this case are not independent; for instance, either or both channels can share their completed elements (e.g., subprocesses) with the other, boosting the recipient's progress toward the requisite amount.

We first test for Sz congruency facilitation through a parametric UCIP structure, specifically a simultaneous Erlang system. Other facilitation mechanisms then are briefly considered, followed by the application of distribution-general assessments of system capacity. The latter include *C*_*OR*_*T*, and related indexes known as the Race-Model Inequality (*RMI*) and Grice Inequality (*GI*; see, e.g., Eidels et al., 2010).

#### Unlimited-capacity, independent parallel process

The simultaneous Poisson process can be used as a parametric expression of an unlimited-capacity, independent parallel architecture (e.g., Townsend, 1984; cf. clinical-science implementations of Carter and Neufeld, 1999). Its distribution analytically provides mean latencies and inter-trial variances of redundant-target trials. It was conjectured that channels of color-only and word-only single-target presentations would simultaneously be operative during redundant-target trials. Because their latencies were nearly identical, color-only and word-only values again were amalgamated for parameter estimation. These estimates were now derived specifically from the Sz data. The value of *v* was 10.00076, and that of *k*′ + *g* was 7, the Sz-isolated values each being correspondingly lower than their counterparts in the 3-group analysis. These parameter values were assigned to each of the simultaneous processes.

The predicted mean for the congruent word-color condition was

(3) $$\begin{array}{r} {E\left(T\right)}_{Stroop,congruent;simultaneous\;Poisson}\;=\\ 2\sum_{s=0}^{k\prime +g-1}\frac{\left(\begin{array}{c} k\prime +g+s-1\\ s \end{array}\right){\left(\frac{1}{2}\right)}^{k\prime +g}{\left(\frac{1}{2}\right)}^{s}\left(k\prime +g+s\right)}{2v} \end{array}$$

Expressed here is *Pr*(*color-channel first-completion*) *E*(*T*|*color-channel first-completion*) + *Pr*(*word-channel first-completion*) *E*(*T*|*word-channel first-completion*); Equation (3) shows that the terms of the left- and right-hand side of this summation are assumed to be identical. By similar reasoning, the predicted variance was

(4) $$\begin{array}{rc} {Var\;\left(T\right)}_{Stroop,\;congruent;\;simultaneous\;Poisson}\\ = & 2\sum_{s=0}^{k\prime +g-1}\left(\begin{array}{c} k\prime +g+s-1\\ s \end{array}\right){\left(\frac{1}{2}\right)}^{k\prime +g}{\left(\frac{1}{2}\right)}^{s}\\ \left(\frac{\left(k\prime +g+s\right)}{{\left(2v\right)}^{2}}\;+\;{\left(\frac{\left(k\prime +g+s\right)}{2v}\right)}^{2}\right)\\ - & {\left(2\overset{k\prime +g-1}{\underset{s=0}{\sum }}\frac{\left(\begin{array}{c} k\prime +g+s-1\\ s \end{array}\right){\left(\frac{1}{2}\right)}^{k\prime +g}{\left(\frac{1}{2}\right)}^{s}\left(k\prime +g+s\right)}{2v}\right)}^{2} \end{array}$$

In Equation (4), each conditional expectation of *T*^2^ is weighted by the probability of the condition (color first or word first), and summed; subtraction of the squared value of the predicted mean yields the predicted variance.

This model seriously under-predicted observations. The predicted mean was 0.553327, vs. the observed 0.664508, and the predicted variance was 0.03383, vs. the observed 0.079147. For Equation (1), the obtained $\chi_{\left(2\right)}^{2}$ was 17.939, and for Equation (2), the $\chi_{\left(20\right)}^{2}$ was 49.791, *p* → 0 in each case. Considering the failure of predictions even at the level of statistical advantage, those of a super-capacity variation can be dismissed out of hand.

#### Gestalt parallel model

In a Gestalt parallel model, redundant targets are merged into a single unit. An Erlang structure again was applied, with *k*′+*g* = 7. A unique Gestalt-rate value *v*_*g*_ was estimated from the Sz color-word—congruent data itself by allowing the color and word targets to be combined into a single unit. As expected, this model's predictions closely fit the empirical data. They nevertheless did not fit better than the predictions that were constructed using parameter values estimated and imported from the Sz group's color-only (≈word only) data. The estimated value of *v*_*g*_ was 9.96925, almost identical to the imported value of 10.00076.

The fit was similarly acceptable in each case. For example, Equation (2)'s Gestalt-model $\chi_{\left(1\right)}^{2}$ was 0.5633, *p* = 0.4599, and that for the color-only (≈word only) imported parameter values was $\chi_{\left(2\right)}^{2}=0.52956$, *p* = 0.7673. A tendered Gestalt model therefore did not improve predictions of the congruent-condition observations over those of the Erlang distribution using the Sz group's color-only (≈word-only) parameter values.

The tests of fit for the Erlang distribution importing the color-only (≈word-only) parameter values, using Equations (1) and (2), were augmented by binning response frequencies according to latency intervals (described in Section Within-Group Performance Homogeneity, above), and computing Pearson χ^2^ and multinomial *G*^2^ (≈χ^2^) values.

Results generalized to these formats of empirical testing, Pearson $\chi_{\left(4\right)}^{2}=1.9339$, *p* = 0.7479; $G_{\left(4\right)}^{2}$ = 1.9303, *p* = 0.7485. Likewise, importing the parameter estimates of the Sz group as obtained in the analysis on all three groups (*k*′ + g = 9, *v* = 12.0699), Pearson $\chi_{\left(4\right)}^{2}=3.4009$, *p* = 0.4391, and $G_{\left(4\right)}^{2}$ = 3.5413, *p* = 0.4716.

The imported parameter values' validity was also probed through parameter-sensitivity analysis. Departure from estimates led to marked elevation in the present statistics. For example, with *v* = 10.00076 (as estimated from the Sz group's color-only [≈word-only] data), and *k*′+ *g* raised from 7 to 10, Pearson $\chi_{\left(4\right)}^{2}$ became 9.09, and $G_{\left(4\right)}^{2}$ rose to 9.356.

#### Distribution-general indexes

The Sz performance data was further assessed for possible congruent-condition facilitation according to distribution-general measures of system capacity. Three indexes were computed from the binned latency frequencies, described above. The race-model inequality

(*RMI*) is stated as follows:

$$\begin{array}{l} {S\;\left(t\right)}_{congruent}\;-{S\left(t\right)}_{color}\;-\;{S\left(t\right)}_{word}\;+1.0\geq 0 \end{array}$$

where *S*(*t*) is the empirically-estimated survivor function at *t*. Inequality violations (negative values) indicate super-capacity. The *GI*, in turn is

$$MIN[S{\left(t\right)}_{color},S\left(t{)}_{word}\right]-S{\left(t\right)}_{congruent}\geq 0.$$

Violations indicate highly limited capacity. Values for these indexes, along with *C*_*OR*_*T*, are presented for the Sz and HC groups in Table 4.

**Table 4:** **Race-Model Inequality, Grice Inequality, and the Capacity-OR-Coefficient for schizophrenia and healthy control groups in the color-word congruent condition**.

	***t(s)***	**0.4**	**0.8**	**1.2**	**1.6**
SZ	*RMI*	0.023678	0.684039	0.959520	0.976500
	*GI*	0.016722	0.018302	−0.013037	0.000090
	*C_*OR*_T*	0.810699	0.574320	0.466000	0.531270
HC	*RMI*	0.006310	0.697062	1.029344	0.993692
	*GI*	0.003254	−0.003155	−0.003155	0.000000
	*C_*OR*_T*	0.975500	0.606450	0.545700	0.552700

RMI, Race-Model Inequality; GI, Grice Inequality; C_OR_T, Capacity-OR-Coefficient; SZ, schizophrenia; HC, healthy controls.

Changes over intervals of *t* resemble those reported by Eidels et al. (2010). Values of *RMI* increased, and those of *C*_*OR*_*T* tended to decrease. In all, capacity dynamics of trial completion were roughly similar across these groups. There was no evidence of super-capacity for either group.

The index, *C*_*OR*_*T* nevertheless was lower than 1.0 throughout. The mean for the Sz group was 0.596, and that for the HC group was 0.670. Taken together, the *GI* and *C*_*OR*_*T* values indicate that for the HC group, single-channel processing, as expressed under the color-only condition, arguably was transferred to the color-word congruent condition. Considering the structure of *C*_*OR*_*T*, a value exceeding 0.5 would be expected for this group. Unlike the Sz group, targets were not strictly redundant, in that *CI*_*color*_ > *CI*_*word*_ was observed for each bin.

For the Sz group, color or word single-channel processing, as expressed in their color-only ≈ word-only condition data, again is supported. The values of *GI* and *C*_*OR*_*T* together also indicate the possibility that system capacity was divided between the color and word channels in the congruent condition. This possibility is empirically equivalent to single-channel processing, given first-completion termination, and the requisite complement of subprocess completions.

### Formal analysis of possible disproportionate MDD word-color incongruency impairment

The possibility of disproportionate MDD impairment under the incongruent condition also was examined with a more fine-grained analysis. Estimates of *CI* were computed separately for the MDD and HC groups under each bin for the color-only (≈congruent) and incongruent conditions (Table 5).

**Table 5:** **Capacity Indexes for Color-Only (≈congruent) and Incongruent Conditions (***CI***_***color-only*** ≈ ***congruent***_; ***CI***_***incongruent***_) and Capacity Ratios (CR) for the Major Depressive Disorder (MDD) and the Healthy Control (HC) groups**.

	***t*(*s*)**	**0.4**	**0.8**	**1.2**	**1.6**
MDD	*CI_color-only ≈ congruent_*	0.0953	1.6063	3.5501	5.2569
	*CI_incongruent_*	0.0221	0.5056	2.2420	5.0750
HC	*CI_color-only ≈ congruent_*	0.1134	1.8173	4.0540	6.4509
	*CI_incongruent_*	0.0494	0.8570	2.4817	4.3536
MDD	$CR\left(\frac{incongruent}{color-only\;\approx \;congruent}\right)$	0.2320	0.3147	0.6310	0.9654
HC	$CR\left(\frac{incongruent}{color-only\;\approx \;congruent}\right)$	0.4356	0.4712	0.6122	0.6749
Color-only ≈ congruent	$CR\left(\frac{MDD}{HC}\right)$	0.8399	0.8839	0.8757	0.8149
Incongruent	$CR\left(\frac{MDD}{HC}\right)$	0.4285	0.5900	0.9034	1.1657

Also presented in Table 5 are capacity ratios (Wenger and Townsend, 2000). Computations addressed inter-condition ratios, *CI*_*incongruren*_/*CI*_*color-only*≈*congruent*_ or *CR*_*incongruent*/*color-only*≈*congruent*_ for each group. Inter-group ratios, *CI*_*MDD*_/*CI*_*HC*_ (*CR*_*MDD*/*HC*_), were computed separately for the color-only (≈congruent), and the color-word incongruent conditions.

The mean *CR*_*incongruent*/*color-only*≈*congruent*_ for the MDD group was 0.5358, highly similar to that of the HC group's 0.5486, contraindicating a more pronounced MDD incongruity impairment. The mean *CR*_*MDD*/*HC*_ for the color-only (≈congruent) condition was 0.8536, and that for the incongruent condition was 0.7719. Overall, the configuration of *CI* and *CR* values were not out of keeping with an Erlang structure, above, that has a single value of *v*; an incremented subprocess number attending higher encoding load, shared by both groups; and an incremented subprocess number with MDD diagnostic status, occurring to both encoding loads.

### Performance accuracy

Tests on group differences in performance accuracy were negative throughout (Section Data Overview). A more pronounced accuracy reduction under incongruent conditions among Sz participants (Sz impairment), however, has been obtained in selected studies (e.g., Perlstein et al., 1998). Such accuracy differences, where found, are interpretable within the present theoretical formulation of additional subprocesses. This account is presented in Appendix A.1. Performance Accuracy.

Where significant accuracy differences have not been obtained, as in the present case, some investigators (e.g., Barch et al., 2004) have opted for so-called Process-Dissociation analysis of correct response proportions at various times *t* following trial onset (Lindsay and Jacoby, 1994). Using this analysis, group differences signifying Sz deficit purportedly have been revealed with respect to probabilities of color and word completions by a time *t*. It is questionable whether this analysis is coherent with the present developments, and indeed with analytical validity of the Process-Dissociation measurement model altogether. Taken up in Appendix A.2. Evaluation of Process-Dissociation Analysis, these issues contraindicate the use of this analysis in clinical cognitive science (or elsewhere).

### Integration of modeled functional, and hemodynamic response function dynamics

We precede our presentation and discussion of our fMRI findings with an exposition of our model-based measurement strategy, emanating from the above developments. Cognitive-task performance dynamics, and those of the hrf (see Ashby, 2011), proceeds as follows. We first construct a model-informed representation of the dynamical trajectory of Stroop-item processing for each combination of encoding load and diagnostic group. Considered alongside are dynamical aspects of the hrf, as set against the model-estimated trajectories of Stroop-item processing. This combination, in turn, guides our selection of MRI-signal analysis—specifically Psychophysiological Interaction Analysis (Friston et al., 1997; SPM8, http://www.fil.ion.ucl.ac.uk/spm). Differences across encoding load and diagnostic groups in time-series covariance are addressed between a theoretically-specified “seed voxel,” and other (searched) voxels residing within an a-priori determined brain region. This form of MRI-signal treatment is considered advantageous in the present context, for reasons stated below.

**Figure 3:**
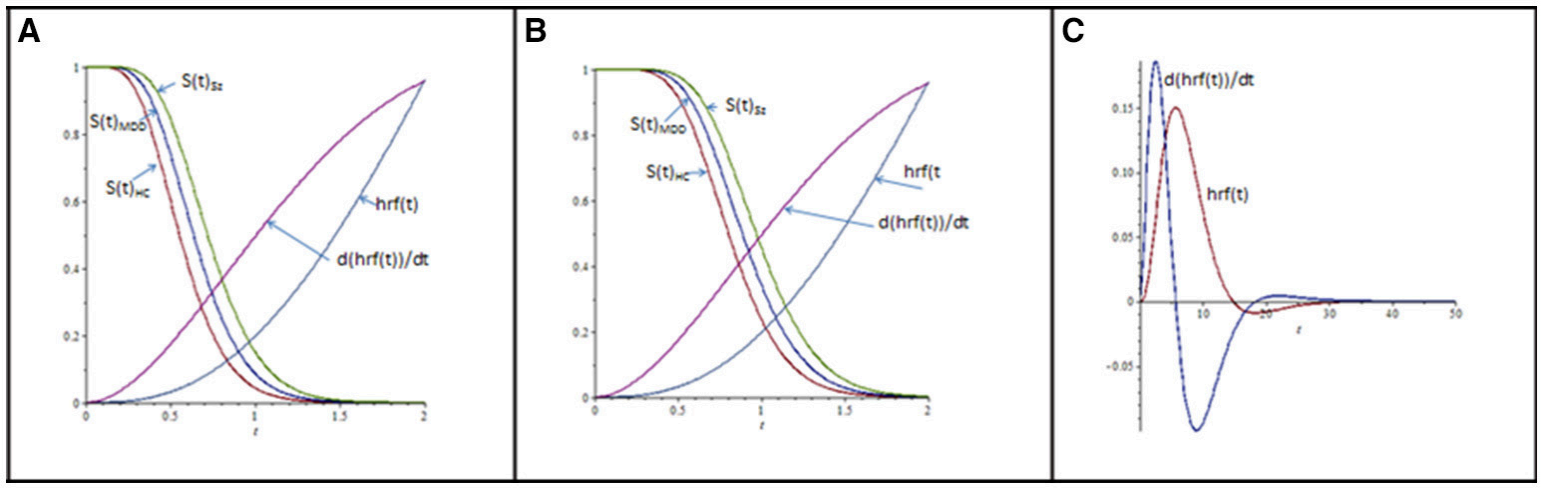
**(A)** Modeled process survivor functions *S*(*t*) for diagnostic groups (abbreviations are as in Figures 1, 2), under low Stroop-item encoding conditions. hrf(*t*) is the hemodynamic response function (of time *t*), modeled as the difference in two gamma-distribution density functions: $\frac{1}{C}\left[\frac{t^{n_{1-1}}}{\lambda_{1}^{n_{1}}\Gamma \left(n_{1}\right)}e^{-\frac{t}{\lambda_{1}}}-a\frac{t^{n_{2-1}}}{\lambda_{2}^{n_{2}}\Gamma \left(n_{2}\right)}e^{-\frac{t}{\lambda_{2}}}\right]$, where *a* = 0.3, *n*_1_ = 4, *n*_2_ = 7, λ_1_ = λ_2_ = 2, and $C=\int_{0}^{50}\left[\cdot \right]\text{d}t$ (Friston et al., 1998; Glover, 1999). **(B)** As in **(A)**, except *S*(*t*) are for higher encoding load. **(C)** Modeled hrf(*t*), and its time derivative d(hrf(*t*))/d*t*, from *t* = 0 to *t* = 50 s, whose extracted 0–2 s segments are inserted into **(A,B)**; hrf(*t*) and d(hrf(*t*))/d*t* are scaled by a constant (=22), for visualization. The time derivative in **(C)** is scaled by *c* = 4.05, for visualization.

When implementing the Erlang distribution (Sections Mathematically Modeled Cognitive Deviation in Sz; Candidate Model Structures; Parameter Estimation and Tests of Empirical Fit), the modeled probability of continued processing over *t* is its survivor function, *S*(*t*). Model values of *S*(*t*) for the three diagnostic groups, under the lower and higher encoding load conditions, are presented in Figures 3A,B. Superposed onto these *S*(*t*) trajectories is a modeled hrf [i.e., hrf(*t*)], along with its time derivative [d(hrf(*t*))/d*t*]. The latter are the 0–2 s segments extracted from the protracted hrf and its derivative, presented in Figure 3C. The juxtaposition of hrf(*t*), d(hrf(*t*))/d*t*, and *S*(*t*) contours indicate methods of choice for MRI data analysis in the present context.

Note first that that the time derivative of hrf(*t*) increases earlier than the hrf(*t*) signal itself. Note as well that the time-series covariance between a seed and searched voxel (*C*_*s, h*; *t*_) exploits this increased temporal resolution. Consider the time derivatives of hrf-estimated seed- and searched-voxel activation, d(hrf(*t*)_*s*_)/d*t*, and, d(hrf(*t*)_*h*_)/d*t*. The quotient [d(hrf(*t*)_*h*_)/d*t*]/[d(hrf(*t*)_*s*_)/d*t*] = d(hrf(*t*)_*h*_)/d(hrf(*t*)_*s*_) expresses the momentary change at *t* of the searched-voxel hrf to that of the seed-voxel hrf. To the degree that their non-linear relations to *t* align with each other, the linear covariance between hrf(*t*)_*h*_ and hrf(*t*)_*s*_ increases, and vice versa. Change in hrf contours elevate more quickly than the contours themselves, promoting the relatively early hrf-covariance estimation of seed-searched voxel association (Neufeld, 2012). More formally,

$$\begin{array}{l} {\left[\int_{0}^{T}f\left(t\right)dt\right]}^{-1}\int_{0}^{T}f\left(t\right)\frac{d\left(hrf{\left(t\right)}_{h}\right)}{d\left(hrf{\left(t\right)}_{s}\right)}dt\\ ={\left[\int_{0}^{T}f\left(t\right)dt\right]}^{-1}E{\left[\frac{d\left(hrf{\left(t\right)}_{h}\right)}{d\left(hrf{\left(t\right)}_{s}\right)}\right]}_{t\in \left[0,T\right]} \end{array}$$

and *C*_*s, h*; *t*_ = *w*{*E* [d(hrf(*t*)_*h*_) / d(hrf(*t*)_*s*_)]_*t*__∈__[0, *T*]_}; here *E* {*} is the expectation of temporal change in the searched voxel to that in the seed voxel over a trial-wise measurement period *T* (=2 s), and *w* is an increasing function.

Bringing forward the estimation of neuro-connectivity, in principle, increases measurement sensitivity to neuro-circuitry associated with targeted processing by stochastically favoring the intersection of high intra-trial process likelihood *S*(*t*) and estimated voxel co-activation. This asset has been of demonstrable value in the context of rapid-processing paradigms in clinical cognitive neuroscience (e.g., Boksman et al., 2005; Neufeld et al., 2010). It squarely is coherent with the goal of selecting cognitive processes *per se* as the events of express interest in event-related fMRI.

### Functional MRI findings

As described in Section Connectivity Estimation and Statistical Criteria, we conducted a search for Stroop-activated regions common to all groups and encoding conditions in the second step of our functional connectivity analysis. Large clusters of significantly activated voxels were found mainly in the parietal lobes (bilaterally), in the dorsal ACC (bilaterally) and in the motor cortex (mostly left sided). Detailed cluster-wise results are presented in Table 6.

**Table 6:** **Local Maxima of statistically significant clusters resulting from Stroop task activation (all groups and stimulus encoding loads combined)**.

**MNI Coord. x,y,z**	**R/L**	**Lobe**	**Gyrus**	**Brodmann area**	**k**	***t*-value (voxel-level)**
10, 16, 38	R	Limbic	Cingulate gyrus	32	532	6.40
−4, 2, 56	L	Frontal	Medial frontal	6	532	9.65
−40, −2, 36	L	Frontal	Precentral gyrus	6	622	7.66
44, 0, 28	R	Frontal	Precentral gyrus	6	111	5.71
−36, 18, 30	L	Frontal	Middle frontal	9	12	5.24
32, −6, 52	R	Frontal	Middle frontal	6	11	5.15
−44, −38, 52	L	Parietal	Inferior parietal lobule	40	4357	11.35
−30, −64, 40	L	Parietal	Precuneus	19	4357	9.70
34, −58, 44	R	Parietal	Inferior parietal lobule	40	791	8.65
34, 20, 4	R	Sub−lobar	Insula	13	194	6.73
−32, 16, 8	L	Sub−lobar	Insula	13	39	5.37

All entries represent an exhaustive list of clusters with p-values reaching FWE-corrected statistical significance p < 0.001.

**Figure 4:**
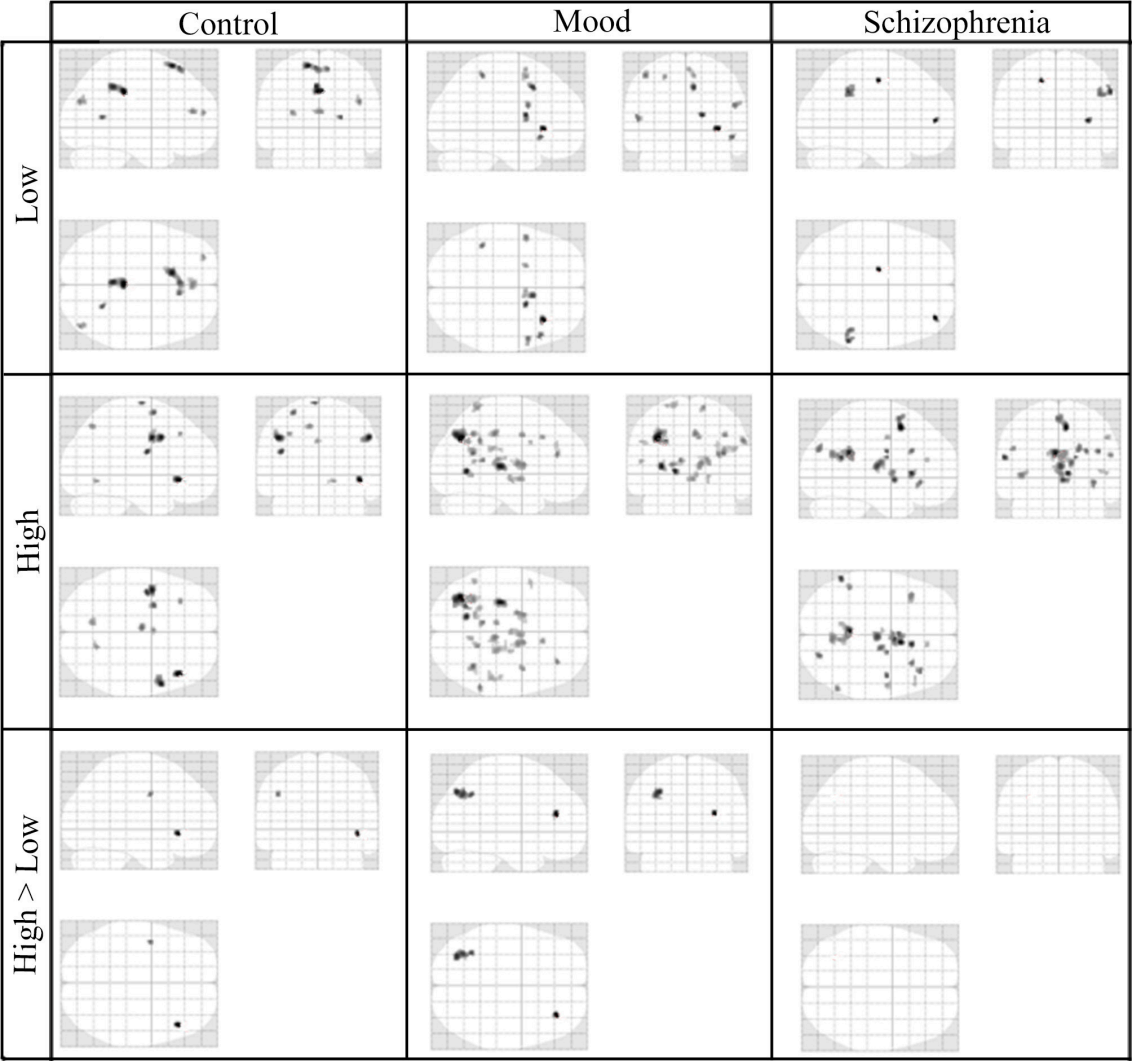
**Glass-Brain Representation of fMRI Data Within-Group tests Results (*k* = 10, *p* < 0.001)**.

**Figure 5:**
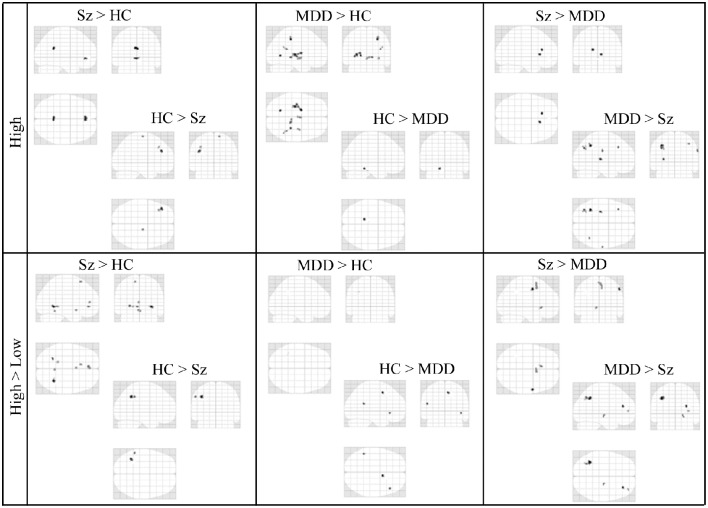
**Glass-Brain Representation of fMRI Data Between-Group tests Results (*k* = 10, *p* < 0.001)**.

In the third step of the analysis, we recruited regions presented in Table 6 to conduct our functional connectivity analysis. Again, our focus is on connectivity and differential connectivity associated with our Sz and control groups and task encoding load, specifically for the seed region located in the dorsal ACC (Broadmann area 32). This area is of particular interest in the cognitive neuroscience of Sz, including that of Stroop performance (Taylor et al., 2015). Figure 4 presents a glass brain representation of the distribution of connectivity clusters for each group and encoding condition, along with directional encoding-load differences within groups. Cluster details are presented in Table S1 of the online supplement. Figure 5 similarly presents the distribution of clusters for group differences under the more challenging high encoding load condition. Also presented are second-order contrasts, addressing patterns of between-group changes in encoding-load differences. Details of these latter contrasts are presented in Table S2 of the online supplement.

## Discussion

### Stroop modeling

A parsimonious account of Stroop-task performance comprises the operation of a single process (color-processing channel). This process is expressed as a parametric Erlang distribution having a single rate parameter, but whose subprocess parameter is incremented under word-color incongruent conditions and with the occurrence of clinical diagnostic status. Cognitive-workload capacity, operationalized as the rate of subprocess transaction, is preserved with Sz, and MDD. Its deployment efficiency, however, is disorder-affected. This account agrees with previous formal modeling of Sz cognition (e.g., Neufeld et al., 2010). The present findings indicate a tenable extension of this parametric combination to MDD (the *p*-values for separate MDD χ^2^'s provided by Equation (2) were 0.49 and 0.55, for lower (color-only ≈ congruent) and higher (incongruent) encoding loads, respectively). The ordering on the subprocess parameter of the HC, MDD, and Sz groups is in keeping with previous findings of their positions in encoding-intensive cognitive performance (e.g., Highgate-Maynard and Neufeld, 1986; George and Neufeld, 1987).

Stroop performance across participants, within groups, was ascertained to be relatively homogeneous. The parallel participant performance profiles allowed for their aggregation, and within-group fixed-parameter status of subsequent modeling (Neufeld and Gardner, 1990; Neufeld, 2007b). The present homogeneity differs from performance on other paradigms, where mixture models have been constructed to accommodate apparent systematic individual differences in parameter values (e.g., Neufeld et al., 2002, 2010). Requirements of such paradigms arguably have gone beyond the current item-feature identification, instead comprising more involved item encoding in the service of memory search. The more basic Stroop-item encoding evidently was less permissive of variability in parameter values across individuals, within groups.

Potentially important innovations to the Stroop task have been introduced by Eidels et al. (2014). In their developments, word reading is ensured by requiring statement of an item's ink color if and only if the carrier word itself is the name of a color (e.g., combination of “yellow” printed in green, but not “mellow” printed in green). The procedure is designed to reduce individual differences in word impingement on processing, and potentially to increase incongruent-condition interference. In the present case, performance nevertheless did not systematically differ across participants within groups or conditions. The incongruity-interference effect also was consistent throughout. Using Eidels et al. (2014) enhanced methodology, however, the latter may become more pronounced. The present modeling interpretation moreover should withstand such paradigmatic variation; clinical groups should be characterized specifically by elevation in encoding subprocesses.

Turning to the color-word congruent condition, for each group, processing could be understood as being the same as that occurring in the color-only (color-only_*Sz*_ ≈word-only_*Sz*_) condition. The result parsimoniously supports the operation of a self-terminating color-target process in the congruent condition (cf. Wenger et al., 2010). Implied is a specific version of the *GI*, namely MIN[*S*(*t*)_*color-only*_, *S*(*t*)_*word-only*_] − *S*(*t*)_*congruent*_ = 0. Values of *GI* approximating 0 are evident in Table 4, for both the Sz and HC groups. Recall, as well, that mean latency can be considered summarily to aggregate *S*(*t*), because $E\left(T\right)=\int_{0}^{\infty }S\left(t\right)dt$, and there was no significant second-order HC-Sz difference in mean latencies involving the congruent, and color-only (color-only_*Sz*_ ≈ word-only_*Sz*_) conditions. Again, the apparent equivalence of the congruent- and color-only (color-only_*Sz*_ ≈ word-only_*Sz*_) condition performance may change with the paradigmatic variation of Eidels et al. (2014).

The modeling results did not support Sz-specific, congruent-condition facilitation. The essential equivalence of color-only and word-only processing, however, was Sz specific. The mechanism(s) of such unique equivalence has yet to be understood. It is possible that these lower-encoding conditions were mutually affected by default-network continuance (Bluhm et al., 2007; Ongür et al., 2010; Williamson and Allman, 2012). Meanwhile, the result nevertheless suggests that neuro-connectivity for this condition amalgam may be different from that of the color-only—congruent amalgam, associated with the HC and MDD groups.

As stated at the outset, the Stroop task has been widely used in clinical-science studies, among various clinical groups. In meta-analytic reviews of cognitive deviation in ADHD, for example, a disproportionate susceptibility to word-feature interference has been noted (Boonstra et al., 2005; Lansbergen et al., 2007). Also observed has been greater across-study heterogeneity of abnormalities, which, contra our findings, may be an extension of within-group heterogeneity.

In their SFT analysis of the Stroop task among non-clinical participants, Eidels et al. (2010) used an incongruent-trial format for their designated single-target condition. Again, this format contrasts that of the current study, where color-only and word-only trials represented the single-target condition. Also, rather than naming the presented color, Eidels et al.'s, (2010) participants indicated the presence-absence of either a target ink-color, or target color name. For example, the color red, and word “red” each might be targets. A single-target incongruent condition could be the word “blue” written in red ink, or the word “red” written in blue ink. The word “red” written in red ink would constitute a double-target condition. Using such combinations of single and double targets, the SFT analysis of Eidels et al. (2010) supported the operation of an independent-channels unlimited capacity (i.e., UCIP) processing architecture.

Our paradigm's incongruent condition corresponds to Eidels et al.'s (2010) ink-color single-target condition. Allowing this condition also to stand as a surrogate for the word single target condition, we examined our performance data for the presence of a UCIP architecture. Treating our incongruent condition this way, results for our HC participants agreed with those found with Eidels et al.'s (2010) non-clinical sample. Like theirs, our *C*_*OR*_*T* values were close to the UCIP-prescribed 1.0 (averaging 0.9631 for latency frequencies binned over intervals appearing in Tables 4, 5). We also tested empirical fit of predictions from a simultaneous Poisson process (as described in Section Unlimited-Capacity, Independent Parallel Process), using Equations (1) and (2), as well as multinomial *G*^2^'s and Pearson χ^2^'s applied to congruent-condition binned frequencies. Results again were uniformly supportive for the HC group (e.g., *p* for Pearson χ^2^ was 0.5687). Results for the Sz and MDD groups, on the other hand, were non-supportive (e.g., Sz's *p* for Pearson χ^2^ = 0.0001). Treating our incongruent trials as conveying a single target, then, an independent-channels unlimited capacity architecture is seen to be viable for our HC group. Such may or may not hold with the use of a bona fide incongruent-format single-target word condition, rather than its current incongruent-format single-target color surrogate.

### MRI findings

We consider first the functional connectivity differences associated with the low and high encoding conditions, for each of the HC, MDD, and Sz groups, presented in Figure 4. In the low-encoding condition, the HC group demonstrated prominent connectivity of the ACC seed region to the posterior cingulate with some connectivity in medial frontal areas. The MDD and Sz groups showed more distributed and smaller clusters of ACC connectivity in the low encoding condition.

In the high encoding condition, the healthy controls demonstrate prominent connectivity in bilateral parietal lobes, with significant clusters of temporal lobe activity. This pattern is in contrast to the functional connectivity observed in the MDD and Sz groups, where the activity is scattered and larger numbers of co-activated clusters are present. The majority of the MDD activity is located in posterior regions, particularly on the left side of the brain, whereas the Sz group demonstrates mostly medial activity, throughout the cingulate and into the thalamus. An increase in the co-activation in the MDD and Sz groups is consistent with the notion of increased subprocesses or constituent operations and less optimal deployment of processing resources. The more encoding intensive incongruent condition elucidates the further elevation of encoding subprocesses present in the diseased state at the neuro-connectivity level of analysis. These observations are consistent with previous findings of more diffuse over-connectivity in the ACC of people with Sz, using word-fluency, and memory-search tasks (Boksman et al., 2005; Boksman, 2006; Neufeld et al., 2010).

It is interesting to note that healthy controls show two areas of statistically greater ACC connectivity when comparing the high stimulus encoding load to the low stimulus encoding load. Such differences between high and low encoding loads persist in the MDD group and involve a more posterior region of the brain. Also of note is the absence of statistically registered differences between ACC connectivity in the high and low encoding conditions in patients with Sz.

Group comparisons under high encoding and second-order differences, comprising changes in high-minus-low encoding-load contrasts across groups, are presented in Figure 5. Although impairments in brain circuitry appear to be present in both the Sz and the MDD groups, the spatial distribution patterns do appear to be different when looking at the within-condition group comparisons (upper panel of Figure 5). Specifically, there is more frontal and prefrontal ACC connectivity in the Sz group compared to the MDD group, and increased but diffuse connectivity with posterior regions of the brain in the MDD group compared to the Sz group.

The second-order differences of encoding-load conditions and groups (lower panel of Figure 5) are considered in light of the other results presented in Figures 4, 5. First, the second-order difference (High-Low Encoding Load)_*Sz*_ - (High-Low Encoding Load)_*HC*_ occurs amidst no statistical ascendancy of high vs. low encoding differences among the Sz participants (Figure 4, lower right frame). By its structure, the second-order difference nevertheless can be positive if the HC group has locations of ACC co-activation that are elevated under low encoding relative to high encoding. Such locations may be selectively attenuated by a network of connectivity recruited specifically to the high encoding condition. In this case, the network evidently also includes high-encoding specific sites, as seen in the reverse second order difference (High-Low Encoding Load)_*HC*_ − (High-Low Encoding Load)_*Sz*_.

This pattern is absent from the MDD control group. First, Figure 5's (High-Low Encoding Load)_*MDD*_ − (High-Low Encoding Load)_*HC*_ comparison indicates no perseveration of low-encoding ACC co-activation with the transition to high encoding relative to the HC group. Second, there is once again evidence of HC-unique recruitment of sites according to the reverse second-order difference.

Results from the second-order contrasts involving the Sz and MDD groups resemble those of the Sz and HC groups, as follows. Certain regions of ACC co-activation under low encoding evidently give way to a different set of regions under high encoding for the MDD group, but perseverate in the Sz group. Altogether, there is “multiple-dissociation” evidence of a reduced separation between low- and high-encoding networks among the Sz group, relative to both the HC and MDD control groups.

Note that, in general, participant motion can raise difficulties with functional connectivity analyses as any departure from the orientation at *t* = 0 (very first image acquisition) could introduce an artifact in the time-courses of signal-intensity at each voxel. Although post-processing methods are available and do help reduce the artifact, it has previously been demonstrated that subject motion is still a significant issue (Power et al., 2012). It may be expected that between the psychiatric conditions and the healthy controls there may be a tendency for decreased (or increased) subject motion. For this reason, the average translational (X, Y, Z) and rotational (pitch, yaw, roll) measures throughout the fMRI acquisition of each individual were compared among groups. No significant differences were observed [Pillai-Bartlett-V approximate *F*_(2, 70)_ = 0.867, *p* = 0.643], suggesting that there was no systematic variation in movement that would be expected to influence the connectivity analysis.

### Model-guided MRI inferences

The presenting picture of Sz neuro-connectivity, paralleling that of parameterized reduction in cognitive encoding efficiency, is one of apparent dis-inhibition of ACC co-activated regions, especially under the higher cognitive encoding demands. The less concentrated pattern of co-activation resembles previous findings, including specific locations of increased spread, notably in medial and frontal and parietal positions (e.g., Boksman et al., 2005; Boksman, 2006; Neufeld et al., 2010).

Part and parcel of the more diffuse co-activation is reduced attenuation under higher encoding demands, of regions co-activated with the ACC under lower encoding demands. Altogether, the comparative neuro-circuitry of the Sz group appears expressly isomorphic with the identified quantitative properties of cognitive performance. Less evident in this group is the strategic deployment of ACC neuro-connectivity, including selective adaptation of networks specifically to encountered encoding conditions.

A consequence of lower encoding efficiency, and its associated neuro-circuitry, is an increase in work-output for completing a given encoding task. The number of discrete events (subprocesses) completed by time *t* for an Erlang distribution is *vt* (e.g., Townsend and Ashby, 1978). The across-trial expected value is

$$\begin{array}{l} {E\;\left(number\;completed\right)}_{{Erlang}_{v}}\;=\;\int_{0}^{\infty }f\left(t\right)vtdt=vE\left(T\right). \end{array}$$

From this perspective, elevated *E*(*T*) implies additional outlay of cognitive work done in Sz to achieve a normal encoding result.

The less channeled neuro-circuitry observed in Sz suggests the possibility of more entropy in their modeled encoding-latency distribution. Computation of Shannon-Weaver entropy was carried out using the probability values for the five bin intervals described above. Shannon-Weaver bits of uncertainty for the HC, MDD, and Sz groups, under the low encoding load, were 1.365, 1.467, and 1.455. Those under the high encoding-load condition were 1.499, 1.586, and 1.640. In this way, the more entropic patterns of ACC connectivity, seen in Figures 4, 5, were co-extensive with quantitatively greater entropy of modeled cognitive performance.

## General discussion

A mandate of clinical cognitive modeling is to broker symptom significance to deviations in functional neuro-circuitry among clinical groups. To that end, a quantitative account of potential symptom significance of elevation in encoding subprocesses, notably for thought-content disorder (delusions and thematic hallucinations), has been developed. Detailed elsewhere (Neufeld, 2007a; Neufeld et al., 2010), the crux of the development is that such encoding elongation disproportionately jeopardizes the input to working memory of cues that signal the objective significance of other successfully encoded material. Without being flanked by contextualizing information, the surviving material is subject to false, if internally coherent, inferences (cf. Yates, 1966; Maher, 1988). The present developments extend formally modeled symptom-significant encoding deficit to perhaps the most widely used selective-attention task in cognitive science, and have ferreted out associated functional neuro-circuitry via high-field MRI. Findings for Sz moreover are separable from those of both healthy and clinical controls, and are founded on a common formal-model platform. Results add to the cognitive neuro-science arsenal on Sz, including recent findings on fMRS of glutamatergic functioning of the ACC (Taylor et al., 2015).

The mental architecture indicated by the selected Erlang distribution of trial latencies is the standard serial (sequential) model of subprocess completion, or a mimicking parallel (concurrent processing) counterpart. The latter include a fixed-capacity parallel model, with reallocation of the processing capacity released by (stochastically) completed subprocesses to those still in progress (Townsend and Ashby, 1983); as well as a first-stage unlimited capacity parallel model (FSUCP, above; Townsend, 1984). Cognitive-behavioral model selection in turn has led to estimation of its functional neuro-circuitry. Illustrated here is the pre-establishing of a prevailing mental architecture, which avoids difficulties of inferring its structure from the very neuro-circuitry being charted (Neufeld, 2007b; cf. Poldrack, 2011).

Identifying encoding elongation, specifically with incremented subprocesses, stands to have certain implications for clinical intervention as follows. The speed of subprocess transaction, *v*, tenably is more aligned with network neuro-dynamics than is the Erlang shape parameter, *k*′, which defensibly relates more to efficiency of their implementation (see Carter and Neufeld, 2007, for neuro-connectionist- model analogs of *v* and *k*′). As such, an increase in *k*′ may signal more ultimate tractability of encoding deficit to therapeutic correction than reduction in *v*.

Furthermore, to intervention, fine-tuned analyses of intra-trial encoding dynamics have been undertaken by Taylor et al. (in press). The additional subprocesses are shown to take their main toll during earlier time windows of processing trials, where the likelihood of successful encoding by Sz participants lags behind that of controls. Interventions depending on information intake thus might exploit the closing of the gap in control-Sz encoding success as the processing opportunity is extended.

The concept of efficiency has had a longstanding role in clinical science (Wishner, 1955). Psychopathology has been characterized in part as an unfavorable ratio of focused to diffuse behavior, analogous to a reduced amount of work accomplished by a machine, relative to its energy consumption. In the current work, this concept has been tied to cognitive neuro-science specifics, including cognitive work transacted, cognitive system entropy, neuro-circuitry diffuseness, and reduced matching of network connectivity to processing conditions.

## Author contributions

RT: research design, neuroimaging and cognitive-behavioral data acquisition and analysis; co-primary responsibility for manuscript preparation. JT: research design; neuroimaging data analysis; manuscript preparation. PW: research design; oversight of participant recruitment; manuscript preparation. MD: neuroimaging signal processing; analysis of neuroimaging summary statistics. RN: mathematical modeling and interpretation of cognitive-behavioral data; co-primary responsibility for manuscript preparation.

### Conflict of interest statement

The authors declare that the research was conducted in the absence of any commercial or financial relationships that could be construed as a potential conflict of interest.

## Footnotes

^1^A review of mathematical modeling of schizophrenia cognition, along with distinctions among analytical (mathematical), computational (with an emphasis on computer simulation), and statistical modeling in clinical science, are available in Neufeld (2015).

^2^An additional mixture model, incorporating geometric and gamma distributions, with similar accommodation of performance patterns tied to incremental subprocesses, has been developed by Cutler (2015) and Cutler and Neufeld (2015).

^3^Note that the present word-only and color-only conditions were not strictly single-targets. Their formats differed from that in their congruent condition (e.g., color “x”'s, vs. color word), which inadvertently may have introduced display features that became incidental distractors with a switch in format (Little et al., 2015).

## Appendix

### A.1. performance accuracy

Additivity of performance latency does not imply additivity of performance accuracy. Let correct responding occur by time *t* whenever the necessary processing for an informed answer has taken place, with its probability defined by the distribution function *F*(*t*). In the absence of such information, 1 − *F*(*t*), a correct response may happen through guessing, with probability *g*. The probability of an accurate response by time *t* thus becomes *F*(*t*) + (1 − *F*(*t*))*g* = *F*(*t*)(1 − *g*) + *g*, and the probability of an error is

(A.1.1) $$\begin{array}{r} 1-F\left(t\right)\left(1-g\right)-g \end{array}$$

With *g* being a constant, Equation (A.1.1) obviously is linear on *F*(*t*).

For an Erlang${}_{v,k\prime }$ structure, *F*(*t*), in turn, is nonlinear on *k*′ (Neufeld and Williamson, 1996). Specifically,

(A.1.2) $$\begin{array}{r} F\left(t\right)\;=\;1-\frac{\Gamma \left(k\prime ,vt\right)}{\Gamma \left(k\prime \right)} \end{array}$$

Section Hazard-Function Considerations. Its second-order difference, ${△}_{k\prime }^{2},F\left(t\right)$, is seen to be negative in regions of *k*′ and *v* representative of the present parameter estimates (e.g., *k*′ = 9, *v* = 11), and notably in the vicinity of the current trial deadlines (e.g., *t* = 1.9 s). Incrementation in subprocesses therefore implies non-additive (potentially, disproportionately greater) error rates alongside additive latencies.

### A.2. evaluation of process-dissociation analysis

In the Process-Dissociation model (PD), the probability of a correct response (i.e., indicating the presented color) in a Stroop color-word congruent condition, at a designated time from trial commencement, is defined as

(A.2.1) $$\begin{array}{r} Pr\left(correct|congruent\right)\equiv Pr\left(color\right)+Pr\left(word\right)\\ -\;Pr\left(word\right)Pr\left(color\right) \end{array}$$

Equation (A.2.1) aligns with an independent race model for a correct response in an OR experimental paradigm (encoding of either the color or the word produces a correct response, the contribution of guessing set aside; see, e.g., Townsend and Wenger, 2004).

In considering this definition further, we define a random variable *T*_*cor*_ as the time at which the participant makes a correct response, where *T*_*cor*_ is set to infinity if the participant does not make a correct response^4^. Then, using notation of a dynamical stochastic process, for a designated finite time of trial progression, *t*, Equation (A.2.1) can be written as

(A.2.2) $$\begin{array}{r} Pr\left(T_{cor}\leq t\right)=F{\left(t\right)}_{color}+F{\left(t\right)}_{word}-F{\left(t\right)}_{color}F{\left(t\right)}_{word} \end{array}$$

where *F*(*t*) is a stochastic-process distribution function.

The PD stated probability of a correct response in a color-word incongruent condition, at a designated time from trial commencement, is

(A.2.3) $$\begin{array}{r} Pr{\left(correct\right)}_{Stroop,incongruent;PD}\equiv Pr\left(color\right)\left(1-Pr\left(word\right)\right) \end{array}$$

Restated in terms of stochastic-process distribution and survivor [*S*(*t*) = 1− *F*(*t*)] functions, Equation (A.2.3) becomes

(A.2.4) $$\begin{array}{r} F{\left(t\right)}_{color}S{\left(t\right)}_{word} \end{array}$$

Equation (A.2.3), hence Equation (A.2.4), putatively provide for the necessity of only color completion by time *t*, whereby responding is said exclusively to be controlled by the color-naming process.

Coherent with a race-model structure implied by Equation (A.2.1), dominance of the color-naming process requires only that the color process be completed before the word process [i.e., color-feature first-passage time; Equation (5) of Khodadadi and Townsend, 2015; cf. (Eidels, 2012)]. Remaining with stochastic-process notation, its race-model probability is

(A.2.5) $$Pr(T_{cor}\leq t)=\int_{0}^{t}f{\left(t\prime \right)}_{\text{color}}S{\left(t\prime \right)}_{word}\text{d}t\prime$$

where the integrand is the density function for color first-completion. Note that in contrast to the density function that appears in Equation (A.2.5), the density function resulting from Equation (A.2.4), obtained as its time derivative, is

$$f\text{ (}t{\text{)}}_{color}S\text{ (}t{\text{)}}_{word}\;-\;f\text{ (}t{\text{)}}_{word}F\text{ (}t{\text{)}}_{color}\text{.}$$

Equation (A.2.3) therefore is mis-specified. Arranging empirical observations according to the PD measurement model results in the following consequences for estimation of *F*(*t*)_*word*_ and of *F*(*t*)_*color*_.

Unlike the distribution function Equation (A.2.5) and that of the Erlang structure (text Sections Mean Diagnostics through Hazard-Function Considerations), each of which monotonically increase on *t*, the expression Equation (A.2.4) first increases and then recedes back to 0 as *S*(*t*)_*word*_ decreases to 0. As with continuous stochastic distribution functions generally, the empirical *Pr*(*T*_*cor*_ ≤ *t*) itself is monotone increasing (e.g., Lindsay and Jacoby's, 1994; Figure 2).

The mis-specification in Equation (A.2.3) ramifies to other expressions in which it participates. Included are estimates of *F*(*t*)_*word*_ and *F*(*t*)_*color*_. By the structures of PD Equations (A.2.1) and (A.2.3), *F*(*t*)_*word*_ becomes Equations (A.2.1) and (A.2.3), estimated as the corresponding difference in empirical congruent- and incongruent-condition values. A result of this subtraction is a *reduction* in PD-based estimation of *F*(*t*)_*word*_ across *t* (as seen in Lindsay and Jacoby's, 1994; Figure 3). Again, this direction of change is contrary to an inevitable stochastic-distribution increase for any continuous distribution function *F*(*t*). More specifically, with Equation (A.2.1) modeling *Pr*(*T*_*cor*_ ≤ *t*)_*congruent*_ and Equation (A.2.3) modeling *Pr*(*T*_*cor*_ ≤ t), positive values of the PD-estimated *F*(*t*)_*word*_, (evident in Lindsay and Jacoby's, 1994; Figure 3), require only that empirical *Pr*(*T*_*cor*_ ≤ *t*)_*congruent*_ > *Pr*(*T*_*cor*_ ≤ *t*)_*incongruent*_, as indeed is seen in their Figure 2. For PD-estimated *F*(*t*)_*word*_ then to actually *decrease* over time, the empirical *Pr*(*T*_*cor*_ ≤ *t*)_*incongruent*_ must increase with *t* more than does the empirical Pr(*T*_*cor*_ ≤ *t*)_*congruent*_, again as is seen in Figure 2. The implausible decrease in estimated *F*(*t*)_*word*_ once more presents itself as a product of a mis-specified measurement model.

Turning to higher estimated *F*(*t*)_*word*_ and lower estimated *F*(*t*)_*color*_ among Sz participants (Barch et al., 2004), such a result is compatible with model mis-specification combined with lower empirical *Pr*(*T*_*cor*_ ≤ *t*)_*incongruent*_. The PD expression of *F*(*t*)_*word*_ as Equations (A.2.1) and (A.2.3), obviously is a decreasing function of Equation (A.2.3). The same combination is sufficient to generate a reduction in estimated *F*(*t*)_*color*_. The PD equations are combined to estimate *F*(*t*)_*color*_ as

$$\begin{array}{l} \frac{1}{1-\left[Pr{\left(T_{cor\leq t}\right)}_{congruent}-Pr{\left(T_{cor\leq t}\right)}_{incongruent}\right]}Pr{\left(T_{cor\;\leq \;t}\right)}_{incongruent}\\ \;=\frac{1}{\frac{1-Pr{\left(T_{cor\leq t}\right)}_{congruent}}{Pr{\left(T_{cor\leq t}\right)}_{incongruent}}+1} \end{array}$$

which is seen to be an increasing function of *Pr*(*T*_*cor*_ ≤ *t*)_*incongruent*._

## References

[B1] Ahn W. Krawitz A. Kim W. Busemeyer J. R. Brown J. (2011). A model-based fMRI analysis with hierarchical Bayesian parameter estimation. J. Neurosci. Psychol. Econ. 4, 95–110. 10.1037/a0020684 23795233 PMC3686299

[B2] Andreasen N. C. (1984a). Scale for the Assessment of Negative Symptoms (SANS). Iowa City, IA: The University of Iowa.

[B3] Andreasen N. C. (1984b). Scale for the Assessment of Positive Symptoms (SAPS). Iowa City, IA: The University of Iowa.

[B4] Aoyama N. Théberge J. Drost D. J. Manchanda R. Northcott S. Neufeld R. W. J.. (2011). Grey matter and social functioning correlates of glutamatergic metabolite loss in schizophrenia. Br. J. Psychiatry 198, 448–456. 10.1192/bjp.bp.110.079608 21628707

[B5] Ashby F. G. (2011). Statistical Analysis of fMRI Data. Cambridge, MA: MIT Press.

[B6] Barch D. M. Carter C. S. Cohen J. D. (2004). Factors influencing Stroop performance in schizophrenia. Neuropsychology 18, 477–484. 10.1037/0894-4105.18.3.477 15291726

[B7] Barlow R. E. Proschan F. (1975). Statistical Theory of Reliability and Life Testing. New York, NY: Holt, Rinehart and Winston Inc.

[B8] Batchelder W. H. (1998). Multinomial processing tree models and psychological assessment. Psychol. Assess. 10, 331–344. 10.1037/1040-3590.10.4.331 12056081

[B9] Batchelder W. H. (2007). Cognitive Psychometrics: Combining Two Psychological Traditions. Amsterdam: CSCA Lecture.

[B10] Batchelder W. H. Riefer D. M. (2007). Using multinomial processing tree models to measure cognitive deficits in clinical populations, in Advances in Clinical Cognitive Science: Formal Modeling of Processes and Symptoms, ed Neufeld R. W. J. (Washington, DC: American Psychological), 19–50.

[B11] Bloxom B. (1984). Estimating response time hazard functions: an exposition and extension. J. Math. Psychol. 28, 401–420.

[B12] Bloxom B. (1985). A constrained spline estimator of a hazard function. Psychometrika 50, 301–321. 10.1007/BF02294107

[B13] Bluhm R. L. Miller J. Lanius R. A. Osuch E. A. Boksman K. Neufeld R. W. J.. (2007). Spontaneous low frequency fluctuations in the BOLD signal in schizophrenic patients: anomalies in the default network. Schizophr. Bull. 33, 1004–1012. 10.1093/schbul/sbm052 17556752 PMC2632312

[B14] Boksman K. (2006). Investigation of Effective Connectivity in Schizophrenia Using Functional Magnetic Resonance Imaging and Sternberg Item Recognition Tasks. Unpublished doctoral thesis, Faculty of Graduate Studies, University of Western Ontario, London, ON.

[B15] Boksman K. Théberge J. Williamson P. Drost D. Malla A. Densmore M.. (2005). A 4.0 Tesla fMRI study of brain connectivity during word fluency in first episode schizophrenia. Schizophr. Res. 75, 247–263. 10.1016/j.schres.2004.09.025 15885517

[B16] Boonstra A. M. Oosterlaan J. Sergeant J. A. Buitelaar J. K. (2005). Executive functioning in adult ADHD: a meta-analytic review. Psychol. Med. 35, 1097–1108. 10.1017/S003329170500499X 16116936

[B17] Busemeyer J. R. Diederich A. (2010). Cognitive Modeling. New York, NY: Sage.

[B18] Carter J. R. Neufeld R. W. J. (1999). Cognitive processing of multidimensional stimuli in schizophrenia: formal modeling of judgment speed and content. J. Abnorm. Psychol. 108, 633–654. 10.1037/0021-843X.108.4.633 10609428

[B19] Carter J. R. Neufeld R. W. J. (2007). Cognitive processing of facial affect: neuro-connectionist modeling of deviations in schizophrenia. J. Abnorm. Psychol. 166, 290–305. 10.1037/0021-843X.116.2.290 17516762

[B20] Cochran W. G. (1957). Analysis of covariance: its nature and uses. Biometrics 13, 261–281. 10.2307/2527916

[B21] Curtis A. T. Gilbert K. M. Klassen L. M. Gati J. S. Menon R. S. (2012). Slice-by-slice B1+ shimming at 7 T. Magn. Reson. Med. 68, 1109–1116. 10.1002/mrm.23319 22213531

[B22] Cutler C. (2015). Addressing Very Short Stimulus Encoding Times in Modeling Schizophrenia Cognitive Deficits. Unpublished master's thesis, Faculty of Graduate Studies, University of Western Ontario, London, ON.

[B23] Cutler C. Neufeld R. W. J. (2015). Addressing very short stimulus encoding times in modeling schizophrenia cognitive deficits, in Paper Presented at Annual Meetings of the Society for Mathematical Psychology (Newport Beach, CA).

[B24] Delucchi K. L. (1993). On the use and misuse of chi-square, in A handbook for Data Analysis in the Behavioral Sciences: Statistical Issues, eds Keren G. Lewis C. (Hillsdale, NJ: Lawrence Erlbaum Associates), 295–320.

[B25] Eidels A. (2012). Independent race of colour and word can predict the Stroop effect. Aust. J. Psychol. 64, 194–198. 10.1111/j.1742-9536.2012.00052.x

[B26] Eidels A. Ryan K. Williams P. Algom D. (2014). Depth of processing in the Stroop task: evidence from a novel forced-reading condition. Exp. Psychol. 61, 385–393. 10.1027/1618-3169/a000259 24836124

[B27] Eidels A. Townsend J. T. Algom D. (2010). Comparing perception of Stroop stimuli in focused versus divided attention paradigms: evidence for dramatic processing differences. Cognition 114, 129–150. 10.1016/j.cognition.2009.08.008 19733840 PMC2812677

[B28] Estes W. K. (1956). The problem of inference from curves based on group data. Psychol. Bull. 53, 134–140. 10.1037/h0045156 13297917

[B29] Evans M. Hastings N. Peacock B. (2000). Statistical Distributions, 3rd Edn. New York, NY: Wiley and Sons.

[B30] Evans S. H. Anastasio E. (1968). Misuse of analysis of covariance when treatment effect and covariate are confounded. Psychol. Bull. 69, 225–234. 10.1037/h0025666 5659655

[B31] First M. Spitzer R. Gibbon M. Williams J. (1997). Structured Clinical Interview (SCID) for DSM-IV Axis 1 Disorders. Washington, DC: American Psychiatric Press Inc.

[B32] Friston K. J. Buechel C. Fink G. R. Morris J. Rolls E. Dolan R. J. (1997). Psychophysiological and modulatory interactions in neuroimaging. Neuroimage 6, 218–229. 10.1006/nimg.1997.0291 9344826

[B33] Friston K. J. Fletcher P. Josephs O. Holmes A. Rugg M. D. Turner R. (1998). Event-related fMRI: characterizing differential responses. Neuroimage 7, 30–40. 10.1006/nimg.1997.0306 9500830

[B34] George L. Neufeld R. W. J. (1987). Attentional resources and hemispheric functional asymmetry in schizophrenia. Br. J. Clin. Psychol. 26, 35–45. 10.1111/j.2044-8260.1987.tb00721.x 3828595

[B35] Gilbert K. M. Curtis A. T. Gati J. S. Klassen L. M. Menon R. S. (2011). A radiofrequency coil to facilitate ${\text{B}}_{1}^{+}$ shimming and parallel imaging acceleration in three dimensions at 7 T. NMR Biomed. 24, 815–823. 10.1002/nbm.1627 21834005

[B36] Glover G. H. (1999). Deconvolution of impulse response in event-related BOLD fMRI. Neuroimage 9, 416–429. 10.1006/nimg.1998.0419 10191170

[B37] Hamilton M. (1959). The assessment of anxiety states by rating. Br. J. Med. Psychol. 32, 50–55. 10.1002/da.22385 13638508

[B38] Hamilton M. A. (1960). A rating scale for depression. J. Neurol. Neurosurg. Psychiatry 23, 56–62. 10.1136/jnnp.23.1.56 14399272 PMC495331

[B39] Highgate-Maynard S. Neufeld R. W. J. (1986). Schizophrenic memory-search performance involving nonverbal stimulus properties J. Abnorm. Psychol. 95, 67–73. 3700850 10.1037//0021-843x.95.1.67

[B40] Johnson S. A. Blaha L. M. Houpt J. W. Townsend J. T. (2010). Systems factorial technology provides new insights on global-local information processing in autism spectrum disorders. J. Math. Psychol. 54, 53–72. 10.1016/j.jmp.2009.06.006 23750050 PMC3676313

[B41] Khodadadi A. Townsend J. T. (2015). On mimicry among sequential sampling models. J. Math. Psychol. 68–69, 37–48. 10.1016/j.jmp.2015.08.007

[B42] Kirk R. E. (2013). Experimental Design: Procedures for the Behavioural Sciences, 4th Edn. New York, NY: Sage.

[B43] Klassen L. M. Menon R. S. (2004). Robust automated shimming technique using arbitrary mapping acquisition parameters (RASTAMAP). Magn. Reson. Med. 51 881–887. 10.1002/mrm.20094 15122668

[B44] Lansbergen M. M. Kenemans J. L. van Engeland H. (2007). Stroop interference and attention-deficit/hyperactivity disorder: a review and meta-analysis. Neuropsychology 21, 251–262. 10.1037/0894-4105.21.2.251 17402825

[B45] Lieberman M. D. Cunningham W. A. (2009). Type I and Type II error concerns in fMRI research: rebalancing the scale. Soc. Cogn. Affect. Neurosci. 4, 423–428. 10.1093/scan/nsp052 20035017 PMC2799956

[B46] Lindsay D. S. Jacoby L. L. (1994). Stroop process dissociations: the relationship between facilitation and interference. J. Exp. Psychol. Hum. Percept. Perform. 20, 219–234. 10.1037/0096-1523.20.2.219 8189189

[B47] Link S. W. (1982). Correcting response measures for guessing and partial information. Psychol. Bull. 92, 469–486. 10.1037/0033-2909.92.2.469

[B48] Little D. R. Eidels A. Fific M. Wang T. (2015). Understanding the influence of distractors on workload capacity. J. Math. Psychol. 68–69, 25–36. 10.1016/j.jmp.2015.08.005

[B49] Luce R. D. (1986). Response Times: Their Role in Inferring Elementary Mental Organization. New York, NY: Oxford University Press.

[B50] Macleod C. (2010). Current directions at the juncture of clinical cognitive science: a commentary on the special issue. Appl. Cogn. Psychol. 24, 450–463. 10.1002/acp.1697

[B51] Maher B. (1966). Principles of Psychopathology: An Experimental Approach. New York, NY: McGraw-Hill.

[B52] Maher B. A. (1988). Delusions as the product of normal cognitions, in Delusional Beliefs, eds Oltmanns T. F. Maher B. A. (New York, NY: Wiley), 333–336.

[B53] McGill W. J. Gibbon J. (1965). The general gamma distribution and reaction times. J. Math. Psychol. 2, 1–18. 10.1016/0022-2496(65)90014-3

[B54] Meehl P. E. (1971). High school yearbooks: a reply to Schwartz. J. Abnorm. Psychol. 77, 143–148. 10.1037/h0030750 5156439

[B55] Minzenberg M. J. Laird A. R. Thelen S. Carter C. S. Glahn D. C. (2009). Meta-analysis of 41 functional neuroimaging studies of executive function in schizophrenia. Arch. Gen. Psychiatry 66, 811–822. 10.1001/archgenpsychiatry.2009.91 19652121 PMC2888482

[B56] Montgomery S. A. Asberg M. (1979). Scale designed to be sensitive to change. Br. J. Psychiatry 134, 382–389. 444788 10.1192/bjp.134.4.382

[B57] Neufeld R. W. J. (2007a). On the centrality and significance of encoding deficit in schizophrenia. Schizophr. Bull. 33, 982–993. 10.1093/schbul/sbm056 17556750 PMC2632328

[B58] Neufeld R. W. J. (2007b). Introduction, in Advances in Clinical Cognitive Science: Formal Modeling and Assessment of Processes and Symptoms, ed Neufeld R. W. J. (Washington, DC: American Psychological Association Publications), 3–18.

[B59] Neufeld R. W. J. (2012). Quantitative clinical cognitive science, cognitive neuroimaging, and tacks to fMRI signal analysis: the case of encoding deficit in schizophrenia, in Paper Presented at the 45th Annual Meeting of the Society for Mathematical Psychology (Columbus, OH).

[B60] Neufeld R. W. J. (2015). Mathematical modeling applications in clinical psychology, in Oxford Handbook of Computational and Mathematical Psychology, eds Busemeyer J. R Townsend J. T. Wang Z. Eidels A. (Oxford: Oxford University Press) 341–368.

[B61] Neufeld R. W. J. Boksman K. Vollick D. George L. Carter J. (2010). Stochastic dynamics of stimulus encoding in schizophrenia: theory, testing, and application. J. Math. Psychol. 54, 90–108. 10.1016/j.jmp.2009.04.002

[B62] Neufeld R. W. J. Carter J. R. Boksman K. Jetté J. Vollick D. (2002). Application of stochastic modelling to group and individual differences in cognitive functioning. Psychol. Assess. 14, 279–298. 10.1037/1040-3590.14.3.279 12214434

[B63] Neufeld R. W. J. Gardner R. C. (1990). Data aggregation in evaluating psychological constructs: multivariate and logical deductive considerations. J. Math. Psychol. 24, 276–296. 10.1016/0022-2496(90)90033-6

[B64] Neufeld R. W. J. McCarty T. (1994). A formal analysis of stressor and stress-proneness effects on basic information processing. Br. J. Math. Stat. Psychol. 47, 193–226. 10.1111/j.2044-8317.1994.tb01034.x 7848874

[B65] Neufeld R. W. J. Williamson P. (1996). Neuropsychological correlates of positive symptoms: delusions and hallucinations, in Schizophrenia: A Neuropsychological Perspective, eds Pantelis C. Nelson H. E. Barnes T. R. E. (London: John Wiley & Sons), 205–235.

[B66] Neufeld R. W. J. Townsend J. T. Jetté J. (2007a). Quantitative response time technology for measuring cognitive-processing capacity in clinical studies, in Advances in Clinical Cognitive Science: Formal Modeling and Assessment of Processes and Symptoms, ed Neufeld R. W. J. (Washington, DC: American Psychological Association), 207–238.

[B67] Neufeld R. W. J. Vollick D. Carter J. R. Boksman K. Levy L. George L. (2007b). A mathematical process account of group and individual differences in memory-search facilitative stimulus encoding, with application to schizophrenia, in Advances in Clinical Cognitive Science: Formal Modeling and Assessment of Processes and Symptoms, ed Neufeld R. W. J. (Washington, DC: American Psychological Association), 147–177.

[B68] Neufeld R. W. J. Vollick D. Highgate S. (1993). Stochastic modelling of stimulus encoding and memory search in paranoid schizophrenia: clinical and theoretical implications, in Schizophrenia: Origins, Processes, Treatment, and Outcome: The Second Kansas Series in Clinical Psychology, eds Cromwell R. L. Snyder R. C. (Oxford: Oxford University Press), 176–196.

[B69] Ongür D. Lundy M. Greenhouse I. Shinn A. K. Menon P. E. Cohen B. M.. (2010). Default mode network abnormalities in bipolar disorder and schizophrenia. Psychiatry Res. 183, 59–68. 10.1016/j.pscychresns.2010.04.008 20553873 PMC2902695

[B70] Peirce J. W. (2007). PsychoPy-Psychophysics software in Python. J. Neurosci. Methods 162, 8–13. 10.1016/j.jneumeth.2006.11.017 17254636 PMC2018741

[B71] Perlstein W. M. Carter C. S. Barch D. M. Baird J. W. (1998). The Stroop task and attention deficits in schizophrenia: a critical evaluation of card and single-trial Stroop methodologies. Neuropsychology 12, 414–425. 10.1037/0894-4105.12.3.414 9673997

[B72] Poldrack R. A. (2011). Inferring mental states from neuroimaging data: from reverse inference to large-scale decoding. Neuron 72, 692–697. 10.1016/j.neuron.2011.11.001 22153367 PMC3240863

[B73] Power J. D. Barnes K. A. Snyder A. Z. Schlaggar B. L. Petersen S. E. (2012). Spurious but systematic correlations in functional connectivity MRI networks arise from subject motion. Neuroimage 59, 2142–2154. 10.1016/j.neuroimage.2011.10.018 22019881 PMC3254728

[B74] Ratcliff R. (1979). Group reaction time distributions and an analysis of distribution statistics. Psychol. Bull. 86, 446–461. 10.1037/0033-2909.86.3.446 451109

[B75] Riefer D. M. Knapp B. Batchelder W. H. Bamber D. Manifold V. (2002). Cognitive psychometrics: assessing storage and retrieval deficits in special populations. Psychol. Assess. 14, 184–201. 10.1037/1040-3590.14.2.184 12056081

[B76] Romans S. E. Tyas J. Cohen M. M. Silverstone T. (2007). Gender differences in the symptoms of major depressive disorder. J. Nerv. Ment. Dis. 195, 905–911. 10.1097/NMD.0b013e3181594cb7 18000452

[B77] Schmitt N. (1996). Uses and abuses of coefficient alpha. Psychol. Assess. 8, 350–353. 10.1037/1040-3590.8.4.350

[B78] Snodgrass J. G. Townsend J. T. (1980). Comparing parallel and serial models: theory and implementation. J. Exp. Psychol. Hum. Percept. Perform. 6, 330–354. 10.1037/0096-1523.6.2.330

[B79] Taylor R. Neufeld R. W. J. Schaefer B. Densmore M. Osuch E. A. Rajakumar N. (2015). Functional magnetic resonance spectroscopy of glutamate in schizophrenia and major depressive disorder: anterior cingulate activity during a color-word Stroop task. Nat. Partner J. Schizophr. 15028, 1–8. 10.1038/npjschz.2015.28 PMC4849454 27336037

[B80] Taylor R. Theberge J. Williamson P. Densmore M. Neufeld R. W. J. (in press). Systems-factorial technology-disclosed stochastic dynamics of Stroop processing in the cognitive neuroscience of schizophrenia, in Systems Factorial Technology: A Theory-Driven Methodology for the Identification of Perceptual Cognitive Mechanisms, eds Fific M. Altieri N. Little D. (New York, NY).

[B81] Tollenaar N. Mooijaart A. (2003). Type I errors and power of the parametric bootstrap goodness-of-fit test: full and limited information. Br. J. Math. Stat. Psychol. 56, 271–288. 10.1348/000711003770480048 14633336

[B82] Townsend J. T. (1984). Uncovering mental processes with factorial experiments. J. Math. Psychol. 28, 363–400. 10.1016/0022-2496(84)90007-5

[B83] Townsend J. T. Ashby F. G. (1978). Methods of modeling capacity in simple processing systems, in Cognitive Theory, Vol. 3, eds Castellan J. Restle F. (Hillsdale, NJ: Erlbaum), 200–239.

[B84] Townsend J. T. Ashby F. G. (1983). Stochastic Modelling of Elementary Psychological Processes. Cambridge: Cambridge University Press.

[B85] Townsend J. T. Fifić M. Neufeld R. W. J. (2007). Assessment of mental architecture in clinical/cognitive research, in Psychological Clinical Science: Papers in Honor of Richard M. McFall, eds Treat T. A. Bootzin R. R. Baker T. B. (Hillsdale, NJ: Erlbaum), 223–258.

[B86] Townsend J. T. Nozawa G. (1995). Spatio-temporal properties of elementary perception: an investigation of parallel, serial, and coactive theories. J. Math. Psychol. 39, 321–359. 10.1006/jmps.1995.1033

[B87] Townsend J. T. Wenger M. J. (2004). The serial-parallel dilemma: a case study in a linkage of theory and method. Psychon. Bull. Rev. 11, 391–418. 10.3758/BF03196588 15376788

[B88] Ungar L. Nestor P. G. Niznikiewicz M. A. Wible C. G. Kubicki M. (2010). Color Stroop and negative priming in schizophrenia: an fMRI study. Psychiatry Res. 181, 24–29. 10.1016/j.pscychresns.2009.07.005 19963356 PMC2806188

[B89] Wenger M. J. Negash S. Petersen R. C. Petersen L. (2010). Modeling and estimating recall processing capacity: sensitivity and diagnostic utility in application to mild cognitive impairment. J. Math. Psychol. 54, 73–89. 10.1016/j.jmp.2009.04.012 20436932 PMC2861301

[B90] Wenger M. J. Townsend J. T. (2000). Basic tools for attention and general processing capacity in perception and cognition. J. Gen. Psychol. 127, 67–99. 10.1080/00221300009598571 10695952

[B91] Wilcox R. R. (1997). Some practical reasons for reconsidering the Kolmogorov-Smirnov test. Br. J. Math. Stat. Psychol. 50, 9–20. 10.1111/j.2044-8317.1997.tb01098.x

[B92] Williamson P. C. Allman J. M. (2011). The Human Illnesses: Neuropsychiatric Disorders and the Nature of the Human Brain. New York, NY: Oxford University Press.

[B93] Williamson P. C. Allman J. M. (2012). Framework for interpreting functional networks in schizophrenia. Front. Hum. Neurosci. 6:184. 10.3389/fnhum.2012.00184 22737116 PMC3380255

[B94] Wishner J. (1955). The concept of efficiency in psychological health and psychopathology. Psychol. Rev. 62, 69–80. 10.1037/h0048963 14357528

[B95] Woodworth R. S. Schlossberg H. (1954). Experimental Psychology. New York, NY: Holt, Rinehart and Winston.

[B96] Yates A. (1966). Psychological deficit. Annu. Rev. Psychol. 17, 111–144. 10.1146/annurev.ps.17.020166.000551 5322632

[B97] Young R. C. Biggs J. T. Ziegler V. E. Meyer D. A. (1978). A rating scale for mania: reliability, validity and sensitivity. Br. J. Psychiatry 133, 429–435. 10.1192/bjp.133.5.429 728692

