# Supplementary material for: ACC Neuro-over-Connectivity Is Associated with Mathematically Modeled Additional Encoding Operations of Schizophrenia Stroop-Task Performance
Source: Front Psychol. 2016 Sep 16;7:1295. doi: 10.3389/fpsyg.2016.01295 (PMC5025455; doi:10.3389/fpsyg.2016.01295)
Supplement: Supplementary file 1 [file Table1.docx]

**Table S1.** Local Maxima of statistically significant clusters resulting from Stroop task activation within combinations of groups and encoding-loads, and encoding-load differences within groups.

| **MNI Coord.**  **x,y,z** | **R/L** | **Lobe** | **Gyrus** | **Brodmann Area or Subregion** | ***k*** | ***t*-value (voxel-level)** |
| --- | --- | --- | --- | --- | --- | --- |
| *HC Low* | | | | | | |
| 24 -54 12 | R | Limbic | Posterior Cingulate | 30 | 13 | 3.85 |
| -2 -30 38 | L | Limbic | Cingulate Gyrus | 31 | 123 | 4.40 |
| -12 22 66 | L, R | Frontal | Superior Frontal Gyrus | 6 | 111 | 4.25 |
| 0 44 18 | L | Frontal | Medial Frontal Gyrus | 9 | 41 | 3.67 |
| -30 56 16 | L | Frontal | Superior Frontal Gyrus | 10 | 13 | 3.61 |
| 44 -78 28 | R | Temporal | Angular Gyrus | 39 | 31 | 3.71 |
| *HC High* | | | | | | |
| -2 2 36 | L | Limbic | Cingulate Gyrus | 24 | 13 | 3.68 |
| -4 -10 76 | L | Frontal | Superior Frontal Gyrus | 6 | 14 | 3.95 |
| 54 10 40 | R | Frontal | Middle Frontal Gyrus | 9 | 70 | 4.49 |
| -28 2 66 | L | Frontal | Middle Frontal Gyrus | 6 | 23 | 4.06 |
| -44 0 40 | L | Frontal | Middle Frontal Gyrus | 6 | 63 | 4.51 |
| -32 32 44 | L | Frontal | Middle Frontal Gyrus | 9 | 10 | 3.69 |
| 44 28 -4 | R | Frontal | Inferior Frontal Gyrus | 47 | 36 | 4.68 |
| -10 -64 52 | L | Parietal | Precuneus | 7 | 21 | 3.71 |
| 16 -60 -6 | R | Occipital | Lingual Gyrus | 19 | 10 | 3.65 |
| *HC High > Low* | | | | | | |
| 44 28 -4 | R | Frontal | Inferior Frontal Gyrus | 47 | 18 | 4.08 |
| -44 -2 38 | L | Frontal | Precentral Gyrus | 6 | 10 | 3.61 |
| *MDD Low* | | | | | | |
| 8 6 58 | R | Frontal | Superior Frontal Gyrus | 6 | 29 | 3.52 |
| -22 8 56 | L | Frontal | Superior Frontal Gyrus | 6 | 12 | 3.61 |
| 8 14 46 | R | Frontal | Medial Frontal Gyrus | 6, 32 | 28 | 4.06 |
| 50 24 -6 | R | Frontal | Inferior Frontal Gyrus | 47 | 14 | 3.85 |
| 34 26 2 | R | Frontal | Inferior Frontal Gyrus | 47 | 34 | 4.42 |
| 54 10 26 | R | Frontal | Inferior Frontal Gyrus | 44 | 26 | 3.78 |
| -52 8 12 | L | Frontal | Precentral Gyrus | 44 | 22 | 3.79 |
| -44 -40 58 | L | Parietal | Inferior Parietal Lobule | 40 | 18 | 3.79 |
| 18 8 16 | R | Sub-lobar | Caudate | Caudate Body | 17 | 4.16 |
| *MDD High* | | | | | | |
| 6 -30 42 | R | Limbic | Cingulate Gyrus | 31 | 44 | 4.02 |
| -16 -60 4 | L | Limbic | Posterior Cingulate | 30 | 26 | 4.71 |
| -10 -42 14 | L | Limbic | Posterior Cingulate | 29 | 12 | 4.04 |
| 10 22 38 | R | Frontal | Cingulate Gyrus | 32 | 13 | 3.80 |
| 32 38 20 | R | Frontal | Middle Frontal Gyrus | 10 | 26 | 3.94 |
| 38 0 40 | R | Frontal | Middle Frontal Gyrus | 6 | 15 | 3.83 |
| -52 8 34 | L | Frontal | Inferior Frontal Gyrus | 9 | 10 | 3.49 |
| 16 -48 70 | R | Parietal | Postcentral Gyrus | 7 | 16 | 3.78 |
| -18 -52 72 | L | Parietal | Postcentral Gyrus | 7, 5 | 32 | 3.66 |
| 58 -46 36 | R | Parietal | Supramarginal Gyrus | 40 | 20 | 3.95 |
| 50 -32 26 | R | Parietal | Inferior Parietal Lobule | 40 | 35 | 3.63 |
| -36 -66 38 | L | Parietal | Inferior Parietal Lobule | 39, 40 | 229 | 5.04 |
| -52 -52 14 | L | Temporal | Superior Temporal Gyrus | 22 | 10 | 3.65 |
| -18 0 10 | L | Sub-lobar | Lentiform Nucleus | Putamen | 45 | 3.92 |
| 14 -4 12 | R | Sub-lobar | Thalamus | Ventral Anterior, Lateral Nucleus | 73 | 3.89 |
| 20 -28 14 | R | Sub-lobar | Thalamus | Pulvinar | 52 | 3.82 |
| -10 -28 14 | L | Sub-lobar | Thalamus | Pulvinar | 13 | 3.80 |
| -30 -24 8 | L | Sub-lobar | Claustrum |  | 83 | 4.88 |
| 4 -2 -4 | R | Sub-lobar | Hypothalamus |  | 53 | 3.79 |
| 20 -14 -4 | R | Sub-lobar | Lentiform Nucleus | Medial Globus Pallidus | 10 | 3.61 |
| 8 -56 0 | R | Occipital | Lingual Gyrus | 18 | 13 | 3.65 |
| *MDD High > Low* | | | | | | |
| 30 38 20 | R | Frontal | Middle Frontal Gyrus | 10 | 24 | 4.27 |
| -30 -72 42 | L | Parietal | Superior Parietal Lobule | 7 | 105 | 3.99 |
| *SZ Low* | | | | | | |
| 34 50 8 | R | Frontal | Middle Frontal Gyrus | 10 | 13 | 3.76 |
| 60 -44 38 | R | Parietal | Supramarginal Gyrus | 40 | 69 | 3.63 |
| *SZ High* | | | | | | |
| 10 40 20 | R | Limbic | Anterior Cingulate | 32 | 11 | 3.47 |
| 4 4 -2 | R | Limbic | Anterior Cingulate | 25 | 72 | 4.22 |
| -4 -46 26 | L | Limbic | Posterior Cingulate Gyrus | 31 | 227 | 4.44 |
| 8 10 54 | R | Frontal | Superior Frontal Gyrus | 6 | 84 | 4.72 |
| 52 -4 42 | R | Frontal | Precentral Gyrus | 6 | 14 | 3.58 |
| 46 26 8 | R | Frontal | Inferior Frontal Gyrus | 13 | 10 | 3.47 |
| 38 32 -4 | R | Frontal | Inferior Frontal Gyrus | 47 | 22 | 3.92 |
| -50 -46 30 | L | Parietal | Supramarginal Gyrus | 40 | 12 | 3.73 |
| 56 -58 24 | R | Temporal | Superior Temporal Gyrus | 39 | 14 | 3.83 |
| -58 -54 22 | L | Temporal | Supramarginal Gyrus | 40 | 19 | 4.23 |
| 10 22 6 | R | Sub-lobar | Caudate | Caudate Head | 21 | 4.36 |
| 2 -16 12 | R, L | Sub-lobar | Thalamus | Medial Dorsal Nucleus | 66 | 4.22 |
| 14 -12 16 | R | Sub-lobar | Thalamus | Ventral Lateral Nucleus | 46 | 4.01 |
| 18 -4 22 | R | Sub-lobar | Caudate | Caudate Body | Of 46 | 3.86 |
| -40 22 2 | L | Sub-lobar | Insula | 13 | 36 | 3.83 |
| *SZ High > Low* | | | | | | |
| None |  |  |  |  |  |  |
| All entries represent an exhaustive list of clusters with *p*-values reaching statistical significance with uncorrected *p*<0.001. Some clusters feature more than one local maximum. Clusters with local maxima part of a previously listed cluster are indicated by the label “Of” prior to the cluster size k.  This table contains the cluster details of data represented graphically in Figure 4. | | | | | | |
