# Supplementary material for: ACC Neuro-over-Connectivity Is Associated with Mathematically Modeled Additional Encoding Operations of Schizophrenia Stroop-Task Performance
Source: Front Psychol. 2016 Sep 16;7:1295. doi: 10.3389/fpsyg.2016.01295 (PMC5025455; doi:10.3389/fpsyg.2016.01295)
Supplement: Supplementary file 2 [file Table2.docx]

**Table S2.** Local maxima of statistically significant clusters of group differences in the high encoding-load condition, and second-order differences of encoding load and groups.

| **MNI Coord.**  **x,y,z** | **R/L** | **Lobe** | **Gyrus** | **Brodmann Area or Subregion** | ***k*** | ***t*-value (voxel-level)** |
| --- | --- | --- | --- | --- | --- | --- |
| *High SZ > HC* | | | | | | |
| 0 42 -2 | R, L | Limbic | Anterior Cingulate | 32 | 25 | 3.78 |
| 2 -46 24 | R, L | Limbic | Posterior Cingulate | 23 | 43 | 3.78 |
| *High HC > SZ* | | | | | | |
| 16 -14 70 | R | Frontal | Superior Frontal Gyrus | 6 | 10 | 3.81 |
| -40 38 32 | L | Frontal | Superior Frontal Gyrus | 9 | 37 | 4.53 |
| -36 30 42 | L | Frontal | Middle Frontal Gyrus | 9 | 16 | 3.65 |
| *High MDD > HC* | | | | | | |
| -16 -60 2 | L | Limbic | Posterior Cingulate | 30 | 13 | 4.01 |
| -18 -2 10 | L | Sub-lobar | Lentiform Nucleus | Putamen | 117 | 4.12 |
| 16 -32 -2 | R | Sub-lobar | Thalamus | Pulvinar | 31 | 3.80 |
| 2 -4 -4 | R | Sub-lobar |  | Hypothalamus | 27 | 3.60 |
| 34 -8 -4 | R | Sub-lobar | Claustrum |  | 16 | 3.54 |
| 0 -12 -6 | L | Brainstem | Midbrain | Mammillary Body | Of 27 | 3.45 |
| *High HC > MDD* | | | | | | |
| -14 -38 -16 | L | Cerebellum | Anterior | Culmen | 12 | 4.22 |
| *High SZ > MDD* | | | | | | |
| 10 22 6 | R | Sub-lobar | Caudate | Caudate Head | 22 | 4.19 |
| *High MDD > SZ* | | | | | | |
| -36 26 40 | L | Frontal | Middle Frontal Gyrus | 9 | 10 | 3.67 |
| 64 -18 30 | R | Parietal | Postcentral Gyrus | 1 | 12 | 3.60 |
| 40 -54 48 | R | Parietal | Inferior Parietal Lobule | 40 | Of 12 | 3.54 |
| -36 -50 44 | L | Parietal | Inferior Parietal Lobule | 40 | 28 | 3.90 |
| -36 -68 36 | L | Parietal | Precuneus | 39, 7 | 28 | 3.66 |
| -30 -24 8 | L | Sub-lobar | Claustrum |  | 19 | 3.81 |
| *High > Low, SZ > HC* | | | | | | |
| 34 -52 -2 | R | Limbic | Parahippocampal Gyrus | 19 | 32 | 4.48 |
| -26 -54 0 | L | Limbic | Parahippocampal Gyrus | 30 | 12 | 4.18 |
| -6 44 -2 | L | Limbic | Anterior Cingulate | 32 | 17 | 3.91 |
| -2 46 10 | L | Limbic | Anterior Cingulate | 32 | 19 | 3.72 |
| -2 10 -10 | L | Limbic | Anterior Cingulate | 25 | 12 | 3.69 |
| -18 -40 -2 | L | Limbic | Parahippocampal Gyrus |  | 11 | 3.57 |
| -12 22 64 | L | Frontal | Superior Frontal Gyrus | 6 | 13 | 3.71 |
| 2 -48 -14 | R, L | Cerebellum | Anterior | Culmen | 11 | 3.66 |
| *High > Low, Control > SZ* | | | | | | |
| -40 -52 40 | L | Parietal | Inferior Parietal Lobule | 40 | 39 | 4.28 |
| -56 -42 42 | L | Parietal | Inferior Parietal Lobule | 40 | 15 | 3.86 |
| *High > Low, MDD > HC* | | | | | | |
| None |  |  |  |  |  |  |
| *High > Low, Control > MDD* | | | | | | |
| 8 8 50 | R | Frontal | Medial Frontal Gyrus | 6 | 17 | 3.89 |
| 46 28 -4 | R | Frontal | Inferior Frontal Gyrus | 47 | 10 | 3.71 |
| -50 -46 20 | L | Temporal | Supramarginal Gyrus | 40 | 12 | 3.77 |
| *High > Low, SZ > MDD* | | | | | | |
| 8 10 50 | R | Frontal | Medial Frontal Gyrus | 32 | 55 | 3.65 |
| 4 10 62 | R | Frontal | Superior Frontal Gyrus | 6 | Of 55 | 3.62 |
| 56 0 48 | R | Frontal | Precentral Gyrus | 6 | 28 | 4.34 |
| -8 22 -2 | L | Sub-lobar | Caudate | Caudate Head | 19 | 3.74 |
| *High > Low, MDD > SZ* | | | | | | |
| 30 38 20 | R | Frontal | Middle Frontal Gyrus | 10 | 17 | 4.08 |
| 36 52 8 | R | Frontal | Middle Frontal Gyrus | 10 | 10 | 3.50 |
| -38 -54 40 | L | Parietal | Inferior Parietal Lobule | 40, 7 | 78 | 4.07 |
| 20 -14 -4 | R | Sub-lobar | Lentiform Nucleus | Medial Globus Pallidus | 12 | 3.58 |
| All entries represent an exhaustive list of clusters with *p*-values reaching statistical significance with uncorrected *p*<0.001. Some clusters feature more than one local maximum. Clusters with local maxima part of a previously listed cluster are indicated by the label “Of” prior to the cluster size k.  This table contains the cluster details of data represented graphically in Figure 5. | | | | | | |
